# HLA typing from RNA-Seq sequence reads

**DOI:** 10.1186/gm403

**Published:** 2012-12-22

**Authors:** Sebastian Boegel, Martin Löwer, Michael Schäfer, Thomas Bukur, Jos de Graaf, Valesca Boisguérin, Özlem Türeci, Mustafa Diken, John C Castle, Ugur Sahin

**Affiliations:** 1 TRON - Translational Oncology at the University Medical Center of the Johannes Gutenberg University, Langenbeckstrasse 1, Building 708, 55131 Mainz, Germany; 2 University Medical Center of the Johannes Gutenberg University Mainz, III, Medical Department, Langenbeckstrasse 1, 55131 Mainz, Germany; 3 Ganymed Pharmaceuticals AG, Freiligrathstrasse 12, 55131 Mainz, Germany

## Abstract

We present a method, seq2HLA, for obtaining an individual's human leukocyte antigen (HLA) class I and II type and expression using standard next generation sequencing RNA-Seq data. RNA-Seq reads are mapped against a reference database of HLA alleles, and HLA type, confidence score and locus-specific expression level are determined. We successfully applied seq2HLA to 50 individuals included in the HapMap project, yielding 100% specificity and 94% sensitivity at a *P*-value of 0.1 for two-digit HLA types. We determined HLA type and expression for previously un-typed Illumina Body Map tissues and a cohort of Korean patients with lung cancer. Because the algorithm uses standard RNA-Seq reads and requires no change to laboratory protocols, it can be used for both existing datasets and future studies, thus adding a new dimension for HLA typing and biomarker studies.

## Background

**Figure 1:**
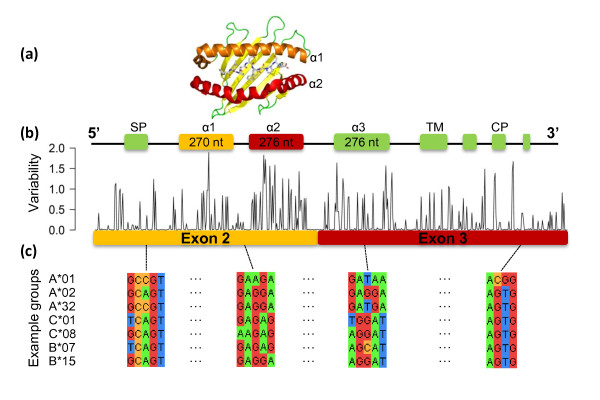
**HLA sequence variability**. **(a) **The helices encoded by exons 2 (α1- chain: orange) and 3 (α2-chain: red) of the HLA class I alleles bind peptides (crystal structure 3OXR from PDB). **(b) **HLA locus is polygenic (A, B, C) and highly polymorphic within and across the three class I genes, showing the nucleotide variability at each position of a multiple sequence alignment of all HLA class I alleles, where 0 and 2 represent minimum and maximum variability, as defined using Shannon's Entropy (2 - Information Content; see Methods). **(c) **Example sequences are shown for four regions in seven HLA groups.

The major histocompatibility complex (MHC) molecules display peptide antigens that are derived from intracellular (class I) and extracellular (class II) proteins on the surface of vertebrate nucleated cells. The human MHC, called the human leukocyte antigen (HLA), is highly polymorphic and comprises three major gene loci for class I (A, B, C) (Figure 1) and three major gene loci for class II (DP, DQ, DR), which are expressed co-dominantly. Each cell expresses three maternal and three paternal HLA class I and three maternal and three paternal class II alpha and beta alleles. Determining the sequence of these molecules, HLA typing, is essential for clinical work (for example, organ transplantation), immune system research, and biomarker and drug development. Current HLA typing techniques use labor- and time-intensive methods, such as sequence-specific oligonucleotide probe (SSOP) hybridization [1], PCR amplification with sequence-specific primers [2], Sanger sequencing [3] and sero-typing [4].

Next generation sequencing (NGS) is a novel platform that enables rapid generation of billions of short nucleic acid sequence reads. Several studies described the use of NGS in high-throughput HLA genotyping using genomic DNA (for examples, see [5,6]). Recently, Lank *et al*. described a method using RNA for high-throughput MHC class I genotyping [7,8], which was applied to assess genotype- and allele-specific expression of MHC class I in human and macaque leukocyte subsets [9]. This method involves the reverse transcription of RNA into cDNA, amplification using highly specific MHC I primers and subsequent bi-directional cDNA amplicon sequencing using Roche/454 GS FLX pyrosequencing, and is able to unambiguously resolve MHC alleles with high accuracy. However, all of these techniques use specialized NGS protocols including primer design to amplify only MHC class I alleles and amplicon sequencing with long reads (≥150 nucleotides) using Roche/454 GS FLX or Illumina GAIIx.

By contrast, gene expression profiling in patient samples using 'whole transcriptome' sequencing (RNA-Seq profiling) typically uses much shorter reads. The adoption of the RNA-Seq platform has been rapid: clinical and research laboratories worldwide have deposited over 14,600 'RNA-Seq' sample profiles into public repositories such as the National Center for Biotechnology Information Sequence Read Archive, including 4,304 human RNA-Seq samples as of 8 October 2012. As opposed to previous methods for determining gene expression, the RNA-Seq platform not only generates expression profiles but the data also contain nucleotide sequence information. Given the large number of RNA-Seq profiles in the public domain and our efforts to develop individualized T cell-mediated cancer vaccines, which require the knowledge of a patient HLA type and HLA expression to prioritize target epitopes [10-12], we sought to develop an algorithm to utilize the sequence content of RNA-Seq reads to determine both HLA type and expression.

To this end, we developed an *in-silico *method, 'seq2HLA', written in python and R, which takes standard RNA-Seq sequence reads in fastq format [13] as input, uses a bowtie index [14] comprising known HLA alleles, and outputs the most likely HLA class I and class II types, a *P*-value for each call, and the expression of each class. As a proof of concept, we applied the method to the recently released 37-nucleotide paired-end RNA-Seq data of 50 Utah residents with ancestry from northern and western Europe (CEU) [15] who are part of the HapMap project and have been previously typed using the PCR-SSOP method [16]. Our method, seq2HLA, achieves 100% specificity and 93.5% sensitivity at a *P*-value of 0.1. Sensitivity versus specificity curves show that the method works best using paired-end reads with length at least 37 nucleotides, that allowing one mismatch in the mapping is optimal, and that calls are more sensitive to read length than the number of reads.

## Materials and methods

### Datasets

We used three publically available NGS RNA-Seq datasets. The first dataset comprises RNA-Seq reads from 60 lymphoblastoid cell lines derived from the CEU HapMap individuals of European descent, including 10 million 37-nucleotide paired-end reads per sample (Accession Number [ERA002336]) [15]. The HLA genotypes of 270 HapMap individuals were previously determined by PCR-SSOP [16] and 51 overlap with the RNA-Seq samples. The Illumina Human Body Map 2.0 project (Accession Number [ERA022994]), the second dataset, comprises reads from 16 different tissues from 15 Caucasian- and one African-American donors, with over seven million 50-nucleotide paired-end reads per sample. The third dataset contains 77 lung RNA-Seq profiles from a lung cancer study in un-typed Korean patients and comprises 100 nucleotide paired-end reads per sample (Accession Number [ERP001058]) [17]. The datasets are provided for download in the European Nucleotide Archive at the European Bioinformatics Institute.

### HLA reference sequences

We downloaded 1,635 HLA-A, 2,247 HLA-B and 1,248 HLA-C (October 2011) and 342 HLA-DQA, 1,976 HLA-DQB and 1,023 HLA-DRB (March 2012) nucleotide sequences from the international ImMunoGeneTics/HLA database at the European Bioinformatics Institute (ftp://ftp.ebi.ac.uk/pub/databases/imgt/mhc/hla/). As exons 2 and 3 (class I) and exon 2 (class II) encode for the peptide-binding site and contain most of the polymorphisms (Figure 1), we extracted these sequences as reference sequences for alignments. In order to include reads mapping to the exon boundaries of the sequence encoding for peptide binding and presentation, we added exon 1 (73 nucleotides long) and 75 nucleotides of exon 4 to the class I reference sequences and 75 nucleotides of exons 1 and 3 to the class II reference sequences.

### Mapping of RNA-Seq reads

We mapped the RNA-Seq reads in fastq format [13] against the reference sequences using bowtie [14]. We optimized alignment parameters (below), including the number of allowed mismatches (-v) and the number of reported mappings (-m or -a).

### Variation and grouping within HLA sequences

To quantify and evaluate the polymorphisms of HLA class I alleles, we computed the variability of sequences inter- and intra-allele groups (Figure S1 in Additional file 1). 'HLA groups' were defined at the two-digit resolution (for example, A*01), consisting of all sequences starting with the same two digits. We computed the Hamming distance of each sequence (exons 2 and 3) with all other sequences, in terms of edit distance, and calculated the inter- and intra-group means.

Furthermore, we calculated the variability at each position across exons 2 and 3 using Shannon's entropy and the nucleotide sequence alignments from all HLA class I alleles (Figure 1). We calculated the variability at each position in terms of information content using the binary logarithm formulation, taking into account the observed frequency of each base (A, T, C and G) across alleles. We plotted 2 - information content, such that 0 represents minimum variability (only one nucleotide observed) and 2 represents maximum variability (all four nucleotides equally likely).

### Read length and HLA typing

To evaluate the trade-offs between read length 'f' and HLA typing, we computed the number of unique length oligonucleotides in each HLA sequence. For each locus, we created all possible f-mers of each HLA exon 2 and 3 sequence. These reads were aligned using bowtie to the respective reference sequences (class I or II) using the mapping parameter -m1 (report only unique mappings). To account for the sequencing errors, the parameter -v (allowed mismatches) was varied to allow for zero, one and two mismatches.

### Data quality control

Of the previously typed CEU HapMap individuals, 51 overlapped with the 60 lymphoblastoid cell lines sequenced by Montgomery *et al*. [15]. After comparison of the HLA types determined by us and others with the established pedigrees for the CEU samples, we excluded sample 1382_1 from further analysis because of HLA inheritance inconsistencies. On examining the RNA-Seq seq2HLA calls, an individual (daughter NA12891) had an HLA type for which the HLA-A, -B and -C alleles matched the individual's father (grandfather NA12891) but the remaining alleles did not match the individual's mother (annotated as sample 1382_1, grandmother NA12892) (Figure S2 in Additional file 1). Further investigation showed that seq2HLA-determined HLA types from the RNA-Seq reads were entirely different from the PCR-SSOP results, the only result in the 51 sample dataset with a complete discrepancy; the grandmother-annotated RNA-Seq reads showed expression of Y-chromosome genes, such as *EIF1AY*, a male-specific gene; and results of seq2HLA using RNA-Seq reads from the grandmother from a different experiment [18] matched the PCR-SSOP results and agreed with the pedigree. Thus, the available data strongly suggest that sample 1382_1 from Montgomery *et al*. is not the individual NA12892, and we removed this individual from our analysis, leaving 50 CEU HapMap individuals (in the following named as Montgomery test samples). Further, given the uniqueness of an individual's HLA type, this example demonstrates how seq2HLA can be used for sample annotation quality control, such as in 'tumor versus normal' experiments where both samples should have the same HLA type.

## Results and discussion

Although HLA alleles are highly polymorphic, all sequences across the A, B and C loci differed by less than 70 nucleotides. Exons 2 and 3 encode the peptide-binding groove of HLA class I molecules (Figure 1) and contain the majority of the variation but are nevertheless 87% identical (Figure S1 in Additional file 1). Furthermore, there are few sequences that are unique to a given allele (Table S1 in Additional file 2). The number of reads mapping to a single HLA allele depends on read length: only 49% of major alleles, that is, those alleles from A, B or C locus, comprise a unique tag 37-mer and 67% have a unique tag 100-mer. Allowing one mismatch to account for sequencing errors, only 0.8% and 3.2% of major alleles contain unique 37- and 100-mers, respectively, demonstrating the difficulty in identifying a unique fingerprint for each allele with short NGS reads. Moreover, using RNA rather than DNA has an additional complication and benefit: results reflect not only HLA type but also expression levels. Given these challenges, we nevertheless sought to make an algorithm to determine HLA types and expression from standard RNA-Seq data.

### Creating the seq2HLA workflow

**Figure 2:**
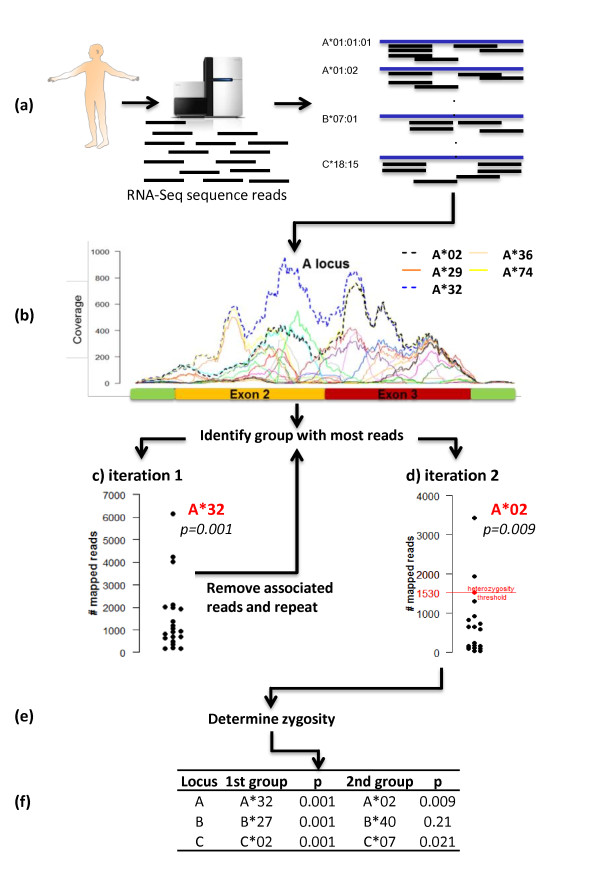
**Workflow of seq2HLA**. **(a) **RNA-Seq reads are mapped against the reference HLA sequences. **(b) **For each locus, the allele with the greatest number of reads is determined. **(c) **Reads associated with the first determined group are removed and the mapping procedure is repeated. **(d) **The second digital haplotype is called. **(e) **Zygosity is determined. **(f) **The genotype and the associated *P*-values are reported.

Seq2HLA works by identifying the HLA alleles associated with the greatest number of NGS RNA-Seq reads. The read-to-HLA mapping and counting is done in two stages (Figure 2). In the first stage, all reads from an RNA-Seq experiment in fastq format are mapped to the reference HLA sequences using bowtie_ENREF_4. For each locus, a 'first round' winning group is chosen. Let $R_{L}^{1}$ denote the set of allelic sequences with respective read counts at locus L∈(A,B,C) in iteration 1. The top-scoring group for locus L is defined as the group that contains the allele with the highest number of mapped reads:

(1) $$a_{R_{L}^{1}}=\text{maxreads}\text{\{}a_{l}|a_{l}\in R_{L}^{1}\}$$

To determine the second group at each locus, the reads mapping to the first round winning group are removed, the remaining reads are mapped against the reference dataset and a 'second round' winning group is selected, again based on the number of mapped reads (Formula 1). At the end of each stage, a 'digital haplotype' is generated.

### Assigning a confidence score

The algorithm assigns a confidence score to each HLA-type call reflecting the likelihood that the called group is correct versus noise. The calculation is based on the assumptions that the right combination of HLA groups will account for the largest number of reads and that the difference between the read count for the right allele combination and the read counts from all other combinations of alleles (the background) reflects the certainty of the HLA-type call.

Given a locus L∈(A,B,C), the distribution $R_{L}^{i}$ of reads mapping to the groups of locus *L *in iteration *i*, and the reads mapping the top-scoring group $a_{R_{L}^{i}}$, let $p\left(a_{R_{L}^{i}}\right)$ denote the probability of observing a value that is greater or equal to $a_{R_{L}^{i}}$ from a normal distribution described by the parameters $mean\left(R_{L}^{i}\right)$ and $sd\left(R_{L}^{i}\right)$:

(2) $$p\left(a_{R_{L}^{i}}\right)=1-pnorm\left(a_{R_{L}^{i}},mean\left(R_{L}^{i}\right),R_{L}^{i}\right)$$

where $pnorm\left(x,y,z\right)$ calculates the cumulative distribution function of a normal distribution centered at y with standard deviation z and returns the probability of observing a value that is equal or larger than x given this distribution. The likelihood of $a_{R_{L}^{i}}$ being a correct HLA call - an outlier from the background - is a *P*-value $p_{outlier}\left(a_{R_{L}^{i}}\right)$ which is calculated according to:

(3) $$p_{outlier}\left(a_{R_{L}^{i}}\right)=pbinom\left(0,x,p\left(a_{R_{L}^{i}}\right)\right)$$

where *x *is equal to the number of elements in $R_{L}^{i}$ and $pbinom\left(q,size,p\right)$ calculates the cumulative distribution function of a binomial distribution with the parameters size (the number of trials) and *P *(probability of success). For quantile q=0, $pbinom\left(q,x,p\left(a_{R_{L}^{i}}\right)\right)$ returns the probability of choosing at least one value that is larger than $a_{R_{L}^{i}}$ in a Bernoulli experiment with *x *trials. Thus, $p_{outlier}$ is a *P*-value reflecting the certainty of the HLA-type call.

Calculation of the confidence score is carried in both iterations of the algorithm in the same way, except for one difference. Whereas, in the first iteration, the number of reads mapping the top-scoring group $a_{R_{L}^{1}}$ is removed from $R_{L}^{1}$, this number $a_{R_{L}^{2}}$ is not removed from $R_{L}^{2}$, resulting in a stricter calculation of the probabilities. The value $a_{R_{L}^{2}}$ now impacts the mean and standard deviation of the distribution, which can result in smaller $p_{outlier}\left(a_{R_{L}^{2}}\right)$ compared with the initial approach.

### Homozygosity score

Within the second iteration, seq2HLA determines whether each locus is homozygous or heterozygous according to the allelic groups typed in the first iteration (Figure 2e). For each locus A, B and C, the median read count across the locus-associated HLA alleles after the first iteration is calculated, which acts as a decision threshold for zygosity. In seq2HLA, we implemented this by simply comparing the number of reads mapping to the second round winning group relative to the median of the first iteration. If the number of reads mapping to the second round winning group was greater than this zygosity decision threshold, the locus was considered heterozygous. If the number was smaller, the locus was called as homozygous. In case of a homozygous prediction, the associated *P*-value reflected the certainty of this locus being homozygous. The smaller the distance between the numbers of reads mapping to the top-scoring group and the median, the more likely this locus was not homozygous. This *P*-value was calculated according to Formula 3, with the modification that the median read count of the first iteration was added to the set of read counts $R_{L}^{2}$ to enable measuring the distance of the top-scoring group to this decision threshold.

Owing to lower sequence variability between the alleles between and within the HLA class II loci as compared with HLA class I loci, the median as the decision threshold was found to be too high because many reads mapped ambiguously in the first iteration. Thus, the optimal threshold was found to be $\frac{median_{L}}{2}$ for locus L∈(DQA,DQB,DRB).

### Expression

HLA expression was determined by the total number of unique sequence reads mapping to class I genes (A, B and C) or class II genes (DQA, DQB and DRB). Owing to the highly conserved nature of the HLA alleles, many reads mapped to multiple alleles and even multiple loci, and thus the assignment between an individual read and an individual allele or locus was frequently ambiguous. To provide an estimate of loci expression, the reads were proportionally assigned to the determined HLA loci based on the mappings between a read and the determined HLA groups (for example, if a read mapped to two different determined HLA loci, each group received a 0.5 count). They were then normalized according to reads per kilobase of exon model per million mapped reads (RPKM) [19] using the actual length of the sub-transcripts contained in the reference dataset: 694 nucleotides for class I and 400 nucleotides, 421 nucleotides and 421 nucleotides for class II (DQA, DQB and DRB respectively).

### Code

Seq2HLA, available as stand-alone and Galaxy modules from http://tron-mainz.de/tron-facilities/computational-medicine/seq2HLA/, is written in python and R. Class I HLA typing using a sample of nine million sequences of 37-nucleotide paired-end reads takes 10 minutes using six central processing units on AMD Opteron 6174.

### Evaluation on healthy individuals

**Figure 3:**
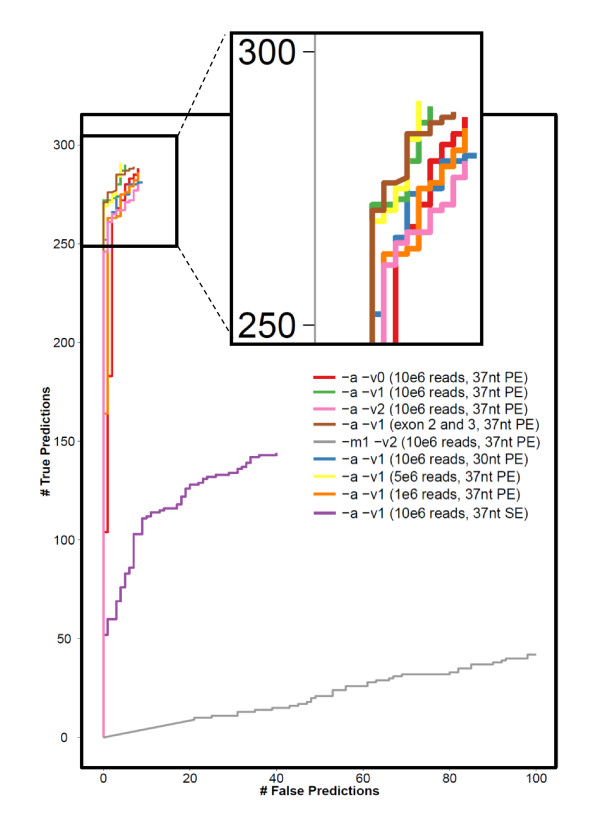
**Sensitivity versus specificity for different mapping and technical parameters applied to the 50 Montgomery test samples**. Bowtie mapping parameters include -a (report all mappings), -m1 (report only unique mappings), -v<0|1|2> (allow zero, one or two mismatches), using the initial reference dataset consisting of exons 1 to 3, plus 75 nucleotides of exon 4. Analysis of different technical parameters comprised varying the average number of reads per sample (1e^6^, 5e^6 ^and 10e^6^) while mapping against the initial reference dataset with '-a -v1', shortening the read length (from 73 nucleotides to 30 nucleotides) and using only one of the read pairs to simulate a single-end read dataset. nt, nucleotide; PE, paired-end; SE, single-end

As a proof of concept, we applied the method to the recently released 37-nucleotide paired-end RNA-Seq data of 50 CEU HapMap individuals (Montgomery test samples) [15]. The 300 MHC class I alleles of these 50 individuals have been previously typed using the PCR-SSOP method [16]. Our method accurately called two-digit HLA types: seq2HLA achieves 100% specificity and 93.5% sensitivity at a *P*-value of 0.1 (Figure 3 and 3Tables S2-5 in Additional file 2).

Out of the 300 alleles, there were five false calls (1.7%), but at *P *>0.1. Not surprisingly, the majority of false calls had high similarities to PCR-SSOP-determined alleles. Individual NA12004 (sample ID 2963_6), for example, was predicted to express B*08 instead of the highly similar B*07, which differs by only 22 of 546 nucleotides (length of exon 2 and 3) (Figure S1c in Additional file 1). Another example was individual NA12006 (sample ID 2992_5), which was predicted to express C*07 instead of C*12, which differ by even fewer nucleotides from each other (15 nucleotides, Figure 2d). Furthermore, one false call, individual NA11993 (sample ID 2005_6), had a missed homozygosity at the B locus, which was due to a high background (high number of ambiguous mapping), with the result that B*40 was assigned a read count higher than the decision threshold. Nevertheless, by assigning a *P*-value to each call that reflects this uncertainty, the user can effectively filter uncertain calls.

Zygosity is challenging for HLA typing with RNA-Seq data: seq2HLA considers the relative number of reads associated with the first- and second-called alleles to determine zygosity. In the Montgomery test samples, 35 of the 150 Class I loci were homozygous: 33 were called correctly by seq2HLA, whereas two cases were falsely classified as heterozygous, with incorrect allele calls but with *P*-values >0.1. For four loci, seq2HLA could not confidently determine zygosity and, rather than make an incorrect HLA call, the loci were classified 'likely homozygous or single allele expressed.' In all cases, the first allele was called correctly; in three cases the second allele was also correct but with too few reads to confidently call the locus heterozygosis and with a high *P*-value assigned, indicating that this locus might be heterozygous with the proposed allele being the second allele.

The samples were also HLA-typed for the class II alleles HLA-DQA, HLA-DQB and HLA-DRB. In the HLA calls by de Bakker et al., 24 of the 300 alleles could not be resolved and thus cannot be evaluated here. Examining seq2HLA class II calls, 264 of 276 are correct (95.7%) and 10 (3.6%) are incorrect, and for two alleles seq2HLA could not make a confident call (Additional file 2, Tables S6-7). 32 of 37 homozygous loci are correctly called. The *P*-values of the class II alleles are less significant overall, particularly in the second iteration, in part due to less sequence variability between alleles of each locus as the variability is primarily encoded only in exon 2 across both alpha and beta loci.

Although the method was validated for HLA class I (A, B, C) and II (DQA, DQB, and DRB) at two-digit resolution, we tested whether the code could be extended to four-digit resolution. The performance of the current algorithm at four-digit class I typing was poor: only roughly 32% of four-digit calls were correct; the incorrect calls typically were highly similar alleles of the same groups with slightly fewer read counts than the true four-digit allele. At this resolution, ambiguous mappings of the short reads were an issue, resulting in poor separation. We expect performance to improve with future datasets containing longer read lengths and additional sequence information outside exons 2 and 3 (class I) and exon 2 (class II) for the reference HLA alleles.

### Comparing different mapping and technical parameters

We used sensitivity versus specificity curves to evaluate the impact on performance of read length, number of reads, paired-end versus single-end and bowtie alignment parameters, such as the number of allowed mismatches, by applying seq2HLA to the Montgomery test samples. Table S2 in Additional file 2 and Figure 3 depict the accuracy using different mapping parameters with respect to allowed mismatches (bowtie options -v0, -v1 and -v2) and reported alignments (bowtie options -m1 -a) for class I. Allowing only unique mappings (-m1) resulted in very poor accuracy with only 51% and 38% correct predictions for zero or two mismatches. Furthermore, it was not possible with these settings to distinguish between homo- and heterozygosity as too many informative reads were lost during the mapping step. The sequencing error rate was 1% to 2%, and thus the optimal number of allowed mismatches for reads with length 37 nucleotides was one (bowtie options -a -v1). As can be seen in Table S2 in Additional file 2, allowing zero or two mismatches (bowtie options -a -v2) resulted in an increase in false predictions.

We further assessed technical parameters, including number of reads, read length and paired-end versus single-end. Figure 3 and Table S4 in Additional file 2 depict the sensitivity versus specificity curves for the different parameters. Decreasing read length from 37 nucleotides to 30 nucleotides rapidly impacted performance, from 93.5% to 89.4% sensitivity at 100% specificity. Decreasing the number of reads had a smaller impact: one million reads per sample in these lymphocyte-derived cell lines still generated HLA calls with 91.6% specificity. The most dramatic performance drop was observed when using single-end reads instead of paired-end, with specificity decreasing from 93.5% sensitivity and 100% specificity to 26.2% sensitivity and 90.5% specificity with the same number of reads. Using only exons 2 and 3 as reference dataset instead of using exons 1, 2, 3 and 75 nucleotides of exon 4 resulted in two more wrong predictions (seven instead of five). In summary, the method works best using paired-end reads with a length at least 37 nucleotides and allowing one mismatch in the mapping, and that calls are more sensitive to read length than the number of reads.

### Further applications

An advantage of seq2HLA is that it can be applied to existing RNA-Seq datasets. We applied seq2HLA to RNA-Seq datasets from the 16 individuals sequenced in the Illumina Human Body Map 2.0 project and nine additional individuals from the CEU HapMap RNA-Seq dataset. None of these individuals had previously been HLA-typed. We report the derived class I and class II HLA types of the nine CEU HapMap individuals in Table S8 in Additional file 2, and of the 16 Illumina Body Map samples in Table 1.

**Table 1:** HLA genotypes of Illumina Human Body Map individuals determined by seq2HLA

HLA class I		HLA-A				HLA-B				HLA-C			
Tissue	Sample Id	A1	*P*	A2	*P*	B1	*P*	B2	*P*	C1	*P*	C2	*P*
**Adipose**	FCB1	A*01	2E-01	A*24	2E-02	B*27	7E-04	B*08	4E-03	C*02	5E-14	C*07	8E-03
**Adrenal**	FCB2	A*25	3E-04	A*03	4E-02	B*18	1E-09	B*40	1E-02	C*02	2E-03	C*12	1E-01
**Brain**	FCB3	A*02	2E-11	homoz	6E-02	B*35	9E-03	B*07	2E-03	C*04	2E-04	C*07	1E-02
**Breast**	FCB4	A*03	1E-02	A*02	4E-04	B*13	5E-09	B*40	3E-03	C*02	3E-03	C*06	4E-02
**Colon**	FCB5	A*01	6E-02	A*03	2E-02	B*08	2E-03	B*13	1E-04	C*06	1E-02	C*07	7E-03
**Heart**	FCB7	A*24	6E-02	A*01	2E-02	B*27	5E-08	B*37	6E-06	C*02	6E-03	C*06	3E-02
**Kidney**	FCB6	A*24	3E-02	A*03	7E-03	B*50	7E-03	B*55	2E-02	C*06	4E-05	C*03	8E-03
**Liver**	FCB8	A*02	3E-09	A*03	5E-03	B*44	2E-11	B*35	1E-03	C*05	1E-01	C*04	3E-02
**Lung**	FCA8	A*24	8E-03	A*11	4E-03	B*81	8E-02	B*07	5E-02	C*04	6E-04	C*07	1E-02
**Lymph node**	FCA7	A*01	4E-04	homoz	5E-02	B*08	4E-07	homoz	6E-01	C*07	2E-13	homoz	1E-04
**Ovary**	FCA3	A*26	4E-02	A*23	4E-02	B*58	3E-06	B*51	1E-01	C*03	9E-03	C*07	9E-03
**Prostate**	FCA6	A*24	5E-04	A*02	5E-04	B*14	3E-05	B*41	3E-03	C*08	2E-01	C*06	3E-02
**Skeletal muscle**	FCA5	A*29	2E-04	A*02	2E-03	B*39	6E-03	B*44	6E-06	C*02	2E-05	C*12	1E-01
**Testes**	FCA2	A*01	5E-02	A*03	3E-02	B*47	1E-03	B*14	5E-04	C*06	3E-05	homoz	2E-04
**Thyroid**	FCA1	A*24	2E-05	A*02	7E-04	B*14	2E-12	B*15	4E-02	C*08	5E-02	C*03	8E-03
**White blood cells**	FCA4	A*25	2E-04	A*03	2E-02	B*07	5E-02	B*40	7E-03	C*03	2E-03	C*07	9E-03

**HLA class II **		**HLA-DQA**				**HLA-DQB**				**HLA-DRB**			
**Tissue**	**Sample Id**	**A1**	***P***	**A2**	***P***	**B1**	***P***	**B2**	***P***	**B1**	***P***	**B2**	***P***

**Adipose**	FCB1	DQA1*05	0E+00	DQA1*03	0E+00	DQB1*02	3E-01	DQB1*03	4E-01	DRB1*03	7E-03	DRB1*11	2E-01
**Adrenal**	FCB2	DQA1*02	6E-02	DQA1*01	3E-01	DQB1*06	3E-02	DQB1*02	3E-01	DRB1*15	3E-02	DRB1*07	1E-02
**Brain**	FCB3	DQA1*03	2E-07	DQA1*01	4E-01	DQB1*05	2E-02	DQB1*03	3E-01	DRB1*01	9E-03	DRB1*04	1E-02
**Breast**	FCB4	DQA1*03	1E-05	DQA1*05	3E-01	DQB1*02	1E-01	DQB1*03	4E-01	DRB1*03	3E-02	DRB1*04	2E-02
**Colon**	FCB5	-	NA	-	NA	DQB1*02	0E+00	homoz	NA	DRB1*03	7E-02	DRB1*07	2E-02
**Heart**	FCB7	DQA1*03	5E-02	DQA1*01	3E-01	DQB1*06	8E-03	DQB1*03	3E-01	DRB1*04	1E-03	DRB1*15	5E-02
**Kidney**	FCB6	DQA1*02	2E-01	DQA1*01	4E-01	DQB1*05	1E-02	DQB1*02	2E-01	DRB1*15	3E-02	DRB1*07	2E-02
**Liver** **Lung**	FCB8 FCA8	DQA1*03 DQA1*04	2E-01 2E-01	DQA1*01 DQA1*01	0E+00 4E-01	DQB1*06 DQB1*06	1E-03 1E-01	homoz DQB1*04	1E+00 4E-01	DRB1*13 DRB1*08	4E-02 1E-01	DRB1*04 DRB1*15	3E-02 3E-02
**Lymph node**	FCA7	DQA1*02	0E+00	homoz	6E-02	DQB1*02	1E-01	DQB1*03	3E-01	DRB1*07	0E+00	DRB1*15	3E-01
**Ovary**	FCA3	DQA1*01	1E-11	DQA1*05	0E+00	DQB1*05	7E-03	homoz	NA	DRB1*10	2E-10	DRB1*12	2E-02
**Prostate**	FCA6	DQA1*03	5E-11	DQA1*05	3E-01	DQB1*02	5E-02	DQB1*03	4E-01	DRB1*04	0E+00	DRB1*15	3E-01
**Skeletal muscle**	FCA5	DQA1*01	0E+00	homoz	NA	DQB1*06	8E-02	DQB1*05	0E+00	DRB1*01	0E+00	DRB1*13	1E-01
**Testes**	FCA2	DQA1*01	0E+00	DQA1*02	4E-01	DQB1*06	0E+00	DQB1*02	0E+00	DRB1*07	2E-02	DRB1*13	3E-01
**Thyroid**	FCA1	DQA1*05	5E-01	DQA1*01	4E-01	DQB1*05	3E-02	DQB1*03	4E-01	DRB1*01	2E-03	DRB1*11	1E-01
**White blood cells**	FCA4	DQA1*01	4E-02	DQA1*05	2E-01	DQB1*05	3E-01	DQB1*03	2E-01	DRB1*12	1E-02	DRB1*01	1E-02

HLA, human leukocyte antigen; NA, no reads detectable.

An RNA-Seq body map dataset consisting of 32 nucleotide single-end reads [20] was previously submitted to the National Center for Biotechnology's Gee Expression Omnibus repository by Wang *et al*. [GEO:GSE12946]. As several of the same tissues are found in both datasets, we internally used them as biological replicates. We applied seq2HLA to nine common tissues samples and found that the HLA types from corresponding tissues, except brain, matched. Thus, eight RNA-Seq profiles from the Wang *et al*. dataset are likely derived from the same samples used in the Illumina Human Body Map 2.0 project and, except brain, should not be used as biological replicates but rather technical replicates.

Seq2HLA does not rely on *a priori *knowledge of population-specific HLA distributions. To demonstrate the applicability of the method to different ethnic groups, we applied seq2HLA to 77 lung RNA-Seq profiles from a lung cancer study in un-typed Korean patients [17]. The HLA calls for each sample are listed in Table S9 in Additional file 2. We compared the distribution of the determined HLA class I alleles from 59 CEU HapMap individuals with European ethnicity (50 Montgomery test samples and the nine previously un-typed CEU HapMap samples), 15 Illumina Body Map samples (those with European ethnicity) and the Korean patients with lung cancer (Table 1 and Tables S3 and S9 in Additional file 2) to established allele frequencies. Encouragingly, we found that the alleles in the 74 CEU HapMap and Illumina Body Map Samples (seq2HLA samples [europ. descent]) are those HLA alleles more frequently found in European populations [21,22] and not frequently found in Korean individuals [21,23,24], such as A*01, A*03, B*08 or C*07 (Figure S3 in Additional file 1). Further, the distribution of alleles found in the Korean patients (Table S9 in Additional file 2) matched the established distribution of alleles in the Korean population; we again found a high correlation between the HLA class I distribution of our predictions and studies assessing HLA class I distribution in the South Korean population (Figure S3 in Additional file 1), including more frequent A*33, B*54 and C*01.

**Figure 4:**
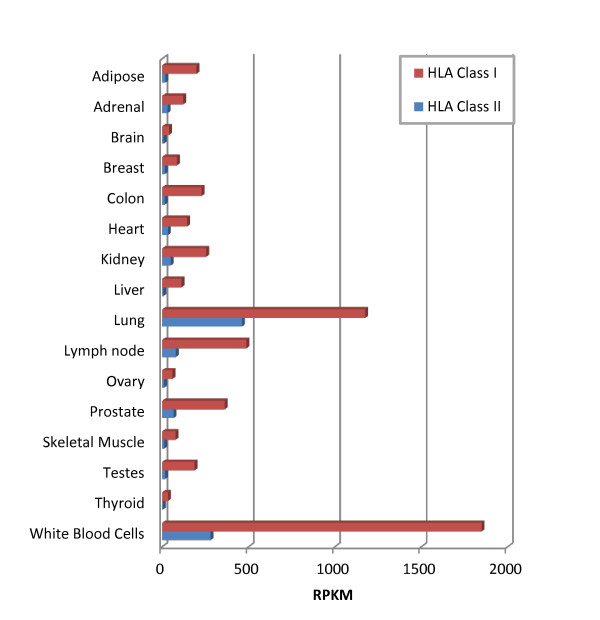
**Summed expression of MHC class I and class II in normal tissues in RPKM units **[19]. Seq2HLA was applied to the 50 nucleotide paired-end RNA-Seq dataset from the Illumina Body Map 2.0 project. HLA, human leukocyte antigen; RPKM, reads per kilobase of exon model per million mapped reads.

Previous RNA-Seq studies using standard algorithms do not accurately determine HLA expression because gene counts determined from read mappings using a single reference genome and transcriptome reflect HLA genetics in addition to HLA expression. By using RNA reads in conjunction with over 6,000 known HLA allele reference sequences, seq2HLA incorporates genetics and expression and outputs HLA expression profiles. We determined HLA class I and II expression across the 16 Illumina Body Map tissues (Additional file 1 and Figure 4), the locus-specific HLA class I and II expression of the 50 Montgomery test samples (Figure S4 in Additional file 1), and locus-specific class I and II expression across the Illumina Body Map tissues (Figure S5 in Additional file 1). As expected, HLA class I expression was highest in the white blood cells and lowest in brain and skeleton muscle [25-29]. Class II expression was highest in lung and leukocytes [30-32]. In contrast to class I molecules, class II expression was expected to be restricted to a subset of cells, the 'professional antigen-presenting cells': indeed, we found lower overall class II expression. In the CEU-derived lymphoblastoid cell lines, the class I loci were expressed at comparable levels, with B slightly higher than A, which is slightly higher than C (Figure S4 in Additional file 1). Class II DRB was expressed at higher levels than DQA and DQB, which were expressed at similar levels. In tissues (Figure S5 in Additional file 1), class I A, B and C were expressed at comparable levels whereas class II DRB was expressed at higher levels than DQA and DQB.

## Conclusions

The main task of the immune system is to protect the body against pathogenic influences from the outside, such as bacteria or viruses, and from the inside, such as tumor-genesis. HLAs play a central role in this task as they present peptides to the immune cells, which are derived either from normal self-proteins in a healthy condition, from pathogens in case of an infection or from abnormal self-proteins.

To determine the HLA type of an individual, common laboratory techniques use either genomic DNA in combination with PCR or proteins and HLA-targeting antibodies. Recent studies employing NGS describe specialized laboratory protocols for high-throughput HLA typing, targeting genomic DNA (for example, see [5,6]) and cDNA [7] from the class I HLA loci. Although these new NGS laboratory protocols and existing clinical testing are critical for clinical applications, such as organ transplantation, an algorithm for use with standard RNA-Seq sequence reads would enable HLA typing of thousands of existing and future samples. Further, none of the existing algorithms determines HLA expression.

The NGS RNA-Seq protocol has been rapidly adopted worldwide to profile gene expression. Here, we show that the sequence reads derived from deep sequencing the transcriptome using standard NGS RNA-Seq can be further used to determine the HLA type and expression. We developed a novel method, seq2HLA, that takes the RNA-Seq read files in fastq format as input and determines the HLA class I and II types and expression. By providing a confidence score to each HLA type, which reflects the likelihood that the called group is correct versus noise, the user can effectively filter results by *P*-value for their specific application. We overcame major challenges when mapping mRNA reads for HLA typing: the HLA genes are not only highly polymorphic but also have thousands of known alleles, many individuals are homozygous at a locus, and most RNA-Seq reads are less than 100 nucleotides long. The seq2HLA achieved 100% specificity and 93.5% sensitivity for two-digit class I prediction on the control samples (Montgomery test samples) and took 10 minutes per sample to run. Applied to RNA-Seq replicates from the same individual, seq2HLA is reproducible, generating identical HLA types. The Illumina Body Map and the Korean lung samples are further demonstrations of the algorithm, showing that the algorithm works in a diverse set of samples.

HLA typing has obvious application to immunological-related processes, from basic research to cohort disease studies to clinical studies. For example, having the HLA type allows the selection of cell lines for use in immunological assays (for example, if we need a non A*02 cell line). A different application is as an inherent sample identifier, enabling determination of incorrect sample annotation. Mislabeled and 'sans papiers' cell lines are not uncommon [33]. Using the HLA type, seq2HLA provides a sample annotation quality control, both for previously un-typed cell lines and for matching cell lines with previously determined HLA types [34]. Further, cancer studies typically compare the transcriptomes of tumor and normal samples from the same individual. Using RNA-Seq data, seq2HLA provides a fast and easy quality control to validate annotation (both tumor and normal should have the same HLA type).

In addition, we find that many non-immunological-aware researchers are currently accumulating large genomic and transcriptomic datasets using NGS, such as with disease population and clinical trial cohorts to find biomarkers (for example, risk factors and response markers). We expect that including HLA types into the analysis will enable the identification of novel HLA disease and response associations. Furthermore, previous RNA-Seq studies do not accurately determine HLA expression as they, during read alignment, do not take into account the polymorphism of the HLA loci. By contrast, seq2HLA not only accurately calls the HLA type of an individual, but also outputs an RPKM HLA expression profile. This is critical for monitoring malignant and therapeutic processes that modify HLA expression.

We foresee extensive synergies and improvements (especially with four-digit-resolution typing) resulting from feedback between seq2HLA and increased NGS read length, increased NGS read accuracy, larger datasets and increased reference HLA sequence databases, especially with more identified and recorded sequence information outside of exons 2 and 3 (class I) and exon 2 (class II).

In summary, we provide a tool that uses standard RNA-Seq reads, requires no change to laboratory protocols, and can be used both for existing and future datasets to determine HLA type and expression.

## Abbreviations

CEU: Utah residents with ancestry from northern and western Europe; HLA: human leukocyte antigen; MHC: major histocompatibility complex; NGS: next generation sequencing; RPKM: reads per kilobase of exon model per million mapped reads; SSO: sequence-specific oligonucleotide

## Competing interests

The authors declare that they have no competing interests.

## Authors' contributions

SB, ML, JCC and US participated in algorithm development, study design and helped draft the manuscript. SB wrote the manuscript, developed the code and performed the analysis. JCC co-wrote the manuscript and supervised and co-developed the algorithms. JG and VB sequenced samples used to develop the algorithm, MS performed HLA genotyping, TB processed NGS sequence reads and sex-specific gene expression analysis, ÖT provided guidance and scientific feedback, and MD had the initial idea. All authors read and approved the final manuscript for publication.

## Supplementary Material

Additional file 1 **Additional figures**. Figure S1: Mean edit distances of all reference sequences (exon 2 and 3 = 546 nucleotides) within and between the groups of alleles to quantify and visualize HLA polymorphisms. Figure S2: Pedigree and HLA types of CEU individuals NA12892, NA12891 and NA12878. Figure S3: Comparison of the distribution of predicted HLA types of this study with population-specific HLA distributions. Figure S4: Average locus-specific expression of HLA class I and II in the 50 Montgomery test samples using seq2HLA. Figure S5: Locus-specific expression of HLA class I and II in the 16 Illumina Human Body Map samples. Click here for file

Additional file 2 **Additional tables**. Table S1: Number of alleles containing at least one f-mer, which is unique for this nucleotide sequence when compared with all alleles within a locus. Table S2: Accuracy of seq2HLA in determining the HLA class I type of the 50 Montgomery test samples using different mapping parameters. Table S3: HLA class I types of the 50 Montgomery test samples. Table S4: Sensitivity versus specificity of different mapping and technical parameters. Table S5: Number of true predictions, false predictions and missed alleles per allelic group. Table S6: Accuracy of seq2HLA in determining the HLA class II type of the 50 Montgomery test samples using the optimal mapping parameter. Table S7: HLA class II types of the 50 Montgomery test samples. Table S8: HLA class I (A) and class II (B) types of nine previously un-typed CEU HapMap individuals. Table S9: Predicted HLA class I types of 77 normal lung derived from Korean individuals. Click here for file
