# Supplementary material for: HLA typing from RNA-Seq sequence reads
Source: Genome Med. 2012 Dec 22;4(12):102. doi: 10.1186/gm403 (PMC4064318; doi:10.1186/gm403)
Supplement: Additional file 1 — Additional figures. Figure S1: Mean edit distances of all reference sequences (exon 2 and 3 = 546 nucleotides) within and between the groups of alleles to quantify and visualize HLA polymorphisms. Figure S2: Pedigree and HLA types of CEU individuals NA12892, NA12891 and NA12878. Figure S3: Comparison of the distribution of predicted HLA types of this study with population-specific HLA distributions. Figure S4: Average locus-specific expression of HLA class I and II in the 50 Montgomery test samples using seq2HLA. Figure S5: Locus-specific expression of HLA class I and II in the 16 Illumina Human Body Map samples. [file gm403-S1.PPTX]

## Slide 1
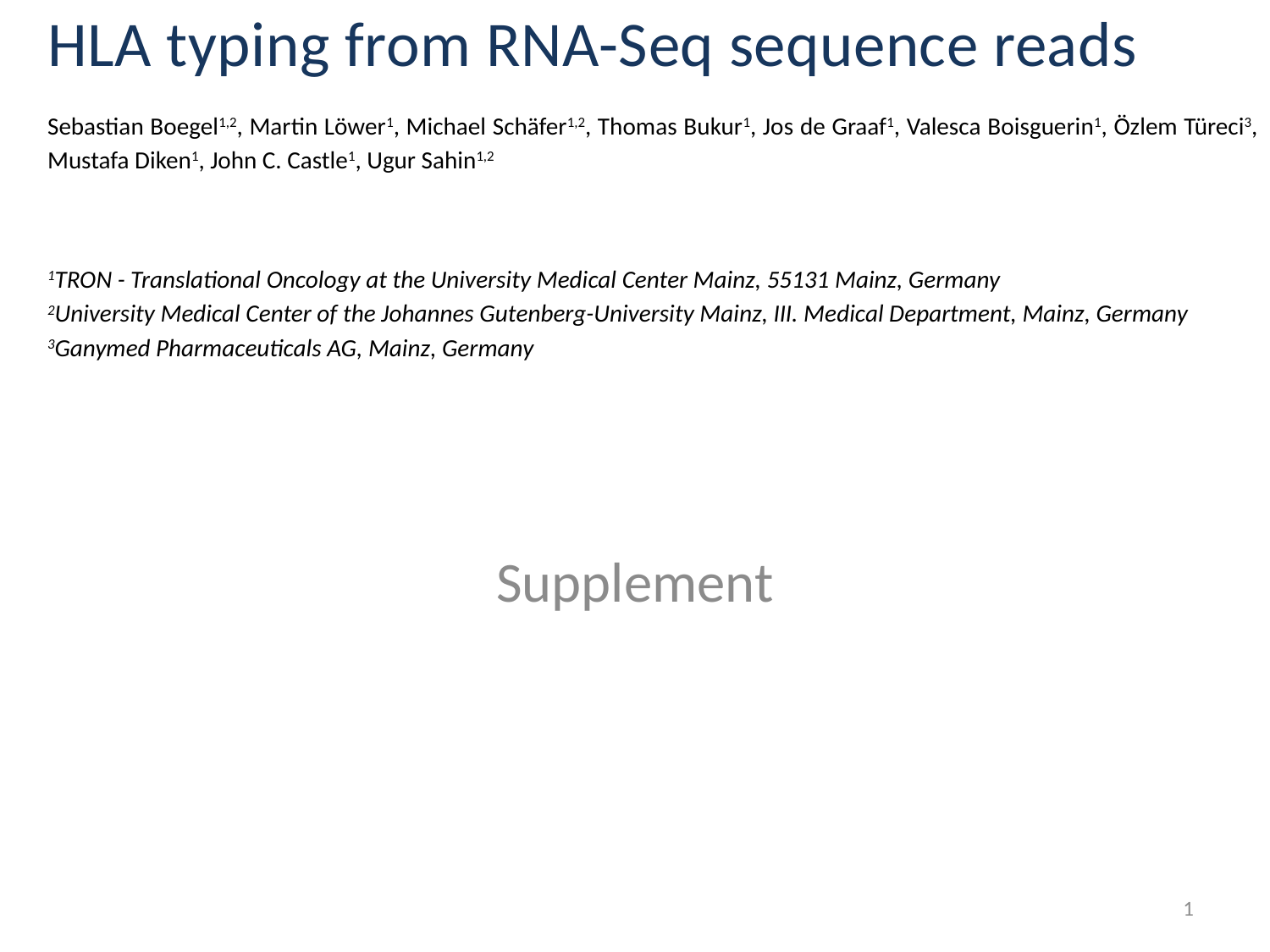

HLA typing from RNA-Seq sequence reads
Sebastian Boegel1,2, Martin Löwer1, Michael Schäfer1,2, Thomas Bukur1, Jos de Graaf1, Valesca Boisguerin1, Özlem Türeci3, Mustafa Diken1, John C. Castle1, Ugur Sahin1,2
1TRON - Translational Oncology at the University Medical Center Mainz, 55131 Mainz, Germany
2University Medical Center of the Johannes Gutenberg-University Mainz, III. Medical Department, Mainz, Germany
3Ganymed Pharmaceuticals AG, Mainz, Germany
Supplement
1

## Slide 2
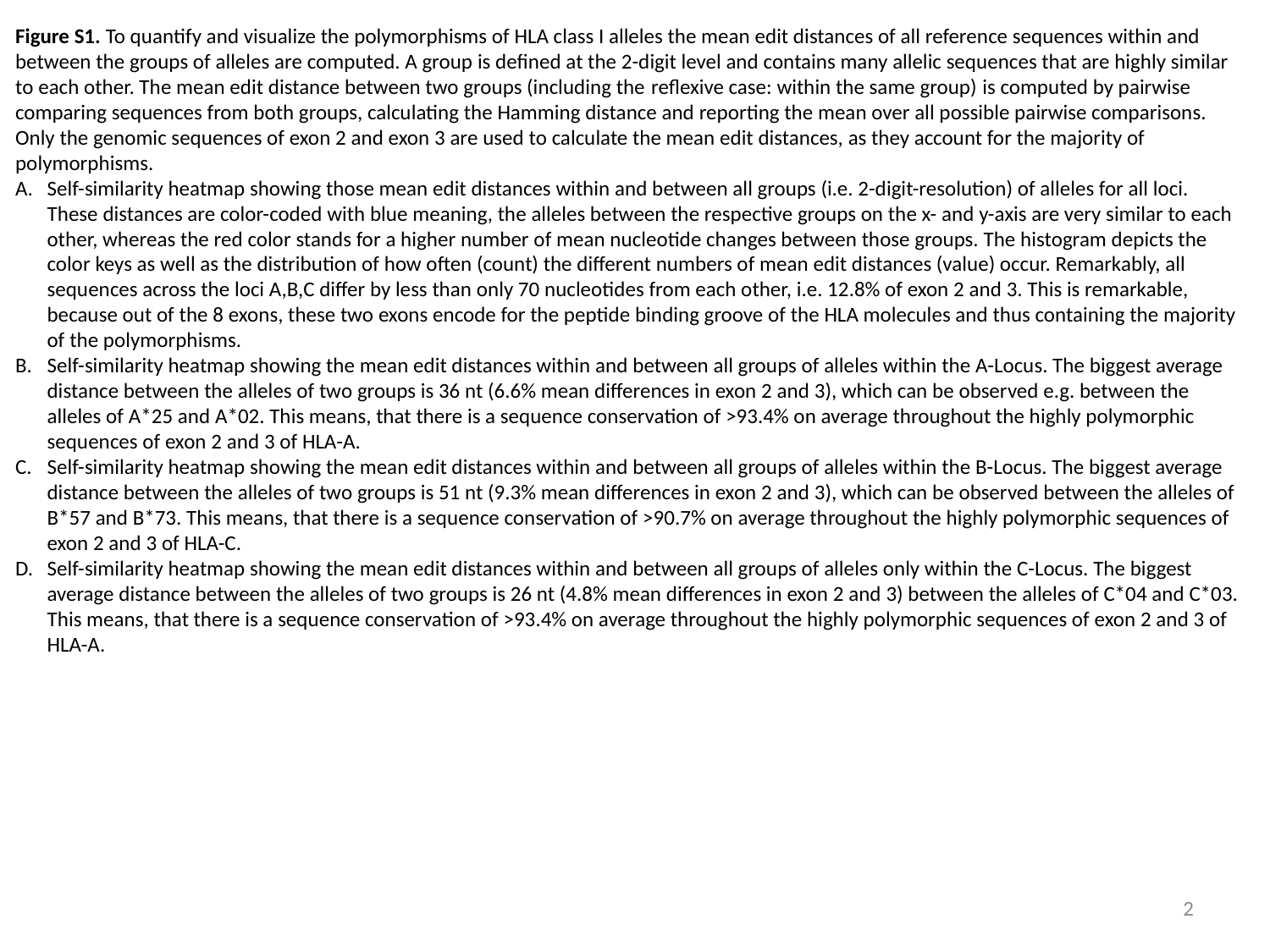

Figure S1. To quantify and visualize the polymorphisms of HLA class I alleles the mean edit distances of all reference sequences within and between the groups of alleles are computed. A group is defined at the 2-digit level and contains many allelic sequences that are highly similar to each other. The mean edit distance between two groups (including the reflexive case: within the same group) is computed by pairwise comparing sequences from both groups, calculating the Hamming distance and reporting the mean over all possible pairwise comparisons. Only the genomic sequences of exon 2 and exon 3 are used to calculate the mean edit distances, as they account for the majority of polymorphisms.
Self-similarity heatmap showing those mean edit distances within and between all groups (i.e. 2-digit-resolution) of alleles for all loci. These distances are color-coded with blue meaning, the alleles between the respective groups on the x- and y-axis are very similar to each other, whereas the red color stands for a higher number of mean nucleotide changes between those groups. The histogram depicts the color keys as well as the distribution of how often (count) the different numbers of mean edit distances (value) occur. Remarkably, all sequences across the loci A,B,C differ by less than only 70 nucleotides from each other, i.e. 12.8% of exon 2 and 3. This is remarkable, because out of the 8 exons, these two exons encode for the peptide binding groove of the HLA molecules and thus containing the majority of the polymorphisms.
Self-similarity heatmap showing the mean edit distances within and between all groups of alleles within the A-Locus. The biggest average distance between the alleles of two groups is 36 nt (6.6% mean differences in exon 2 and 3), which can be observed e.g. between the alleles of A*25 and A*02. This means, that there is a sequence conservation of >93.4% on average throughout the highly polymorphic sequences of exon 2 and 3 of HLA-A.
Self-similarity heatmap showing the mean edit distances within and between all groups of alleles within the B-Locus. The biggest average distance between the alleles of two groups is 51 nt (9.3% mean differences in exon 2 and 3), which can be observed between the alleles of B*57 and B*73. This means, that there is a sequence conservation of >90.7% on average throughout the highly polymorphic sequences of exon 2 and 3 of HLA-C.
Self-similarity heatmap showing the mean edit distances within and between all groups of alleles only within the C-Locus. The biggest average distance between the alleles of two groups is 26 nt (4.8% mean differences in exon 2 and 3) between the alleles of C*04 and C*03. This means, that there is a sequence conservation of >93.4% on average throughout the highly polymorphic sequences of exon 2 and 3 of HLA-A.
2

## Slide 3
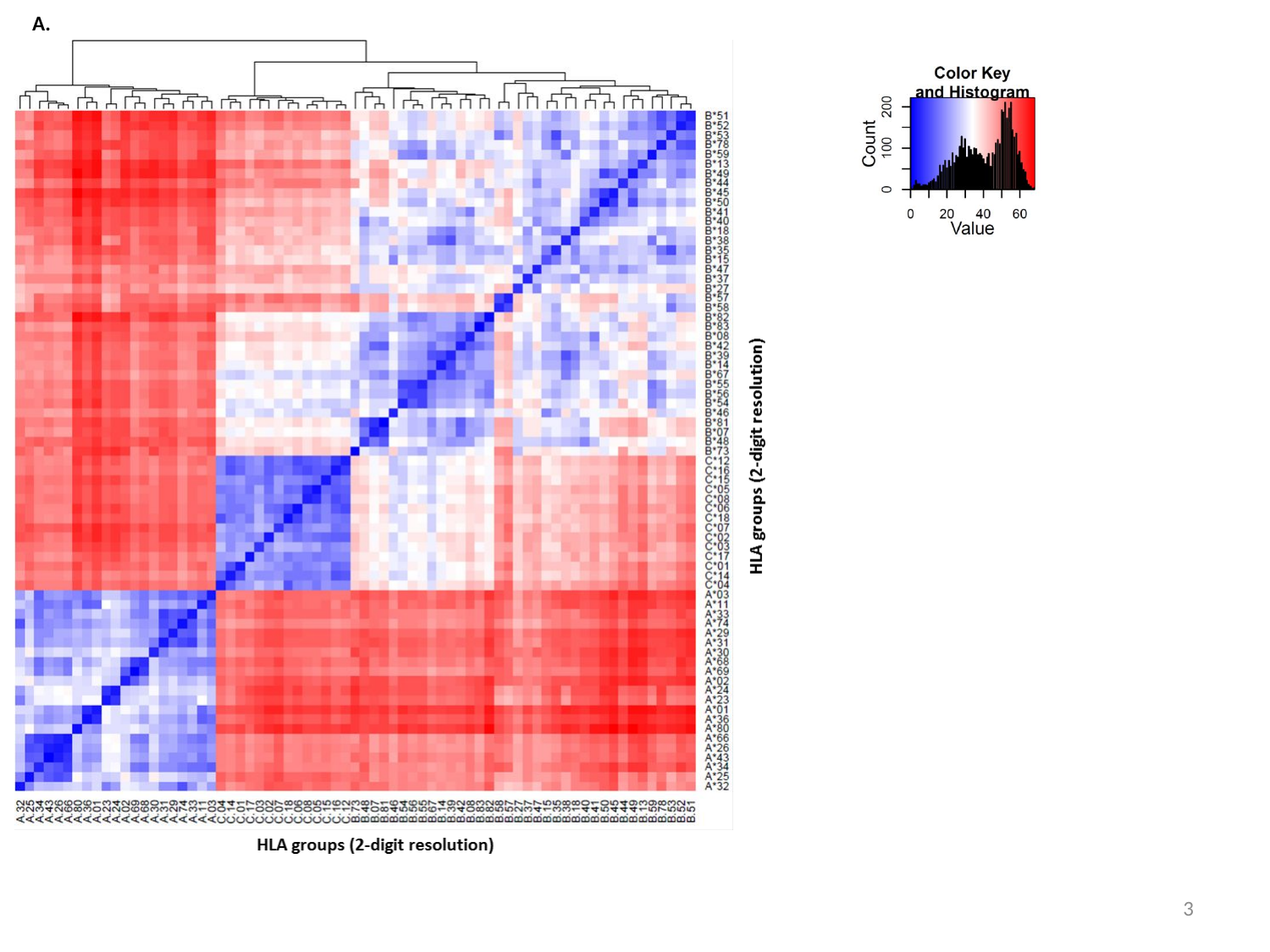

A.
3

## Slide 4
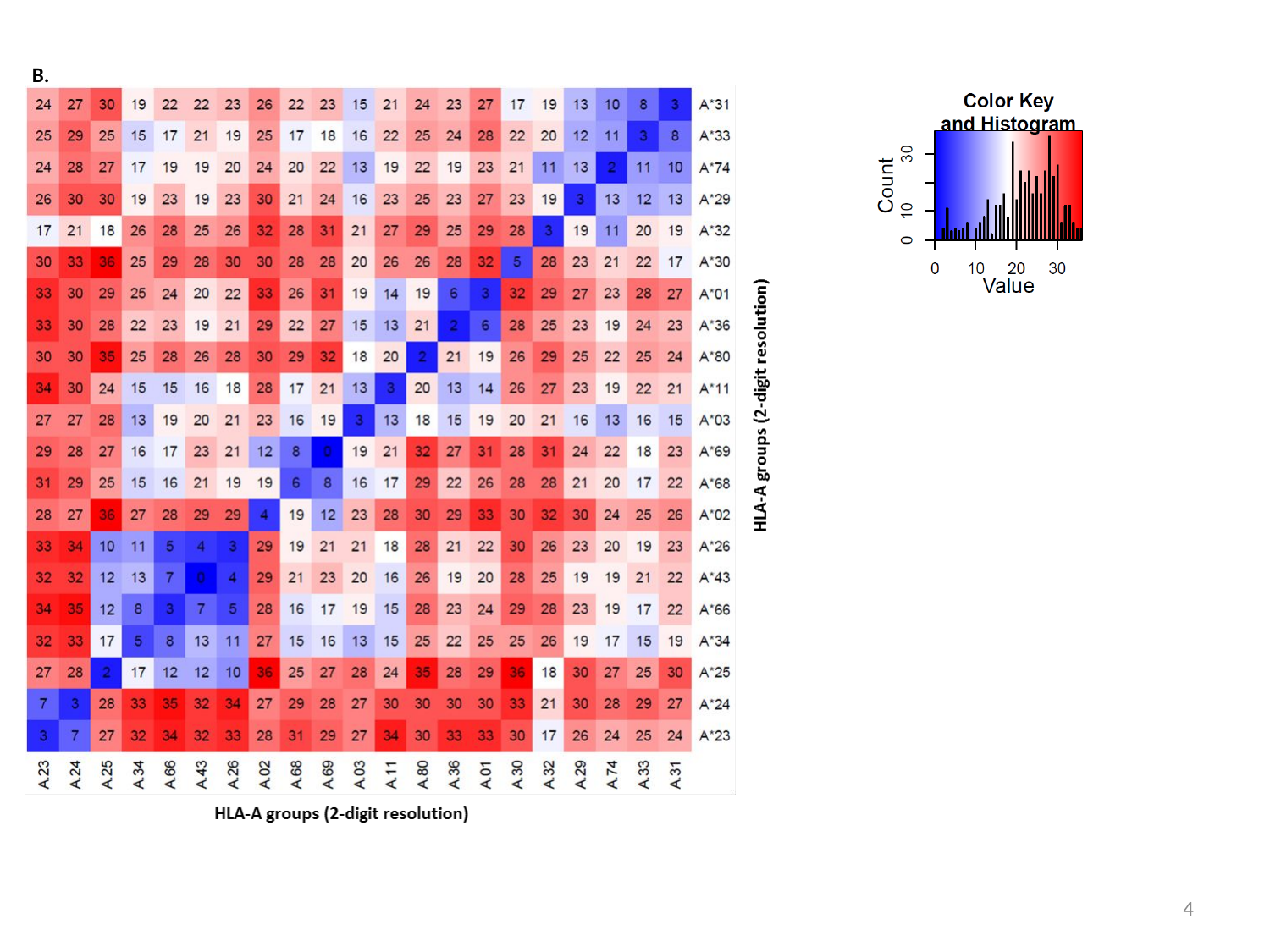

B.
4

## Slide 5
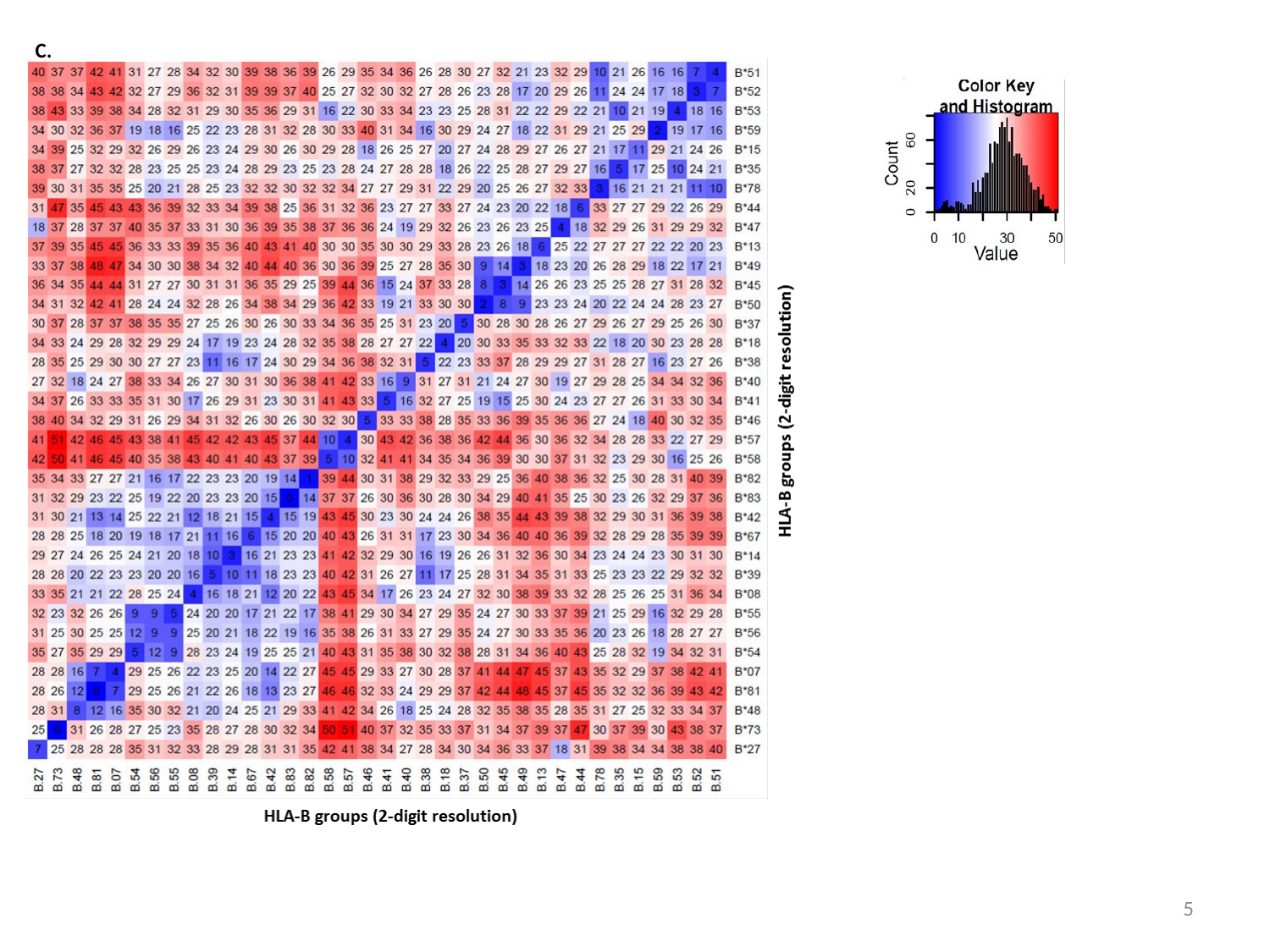

C.
5

## Slide 6
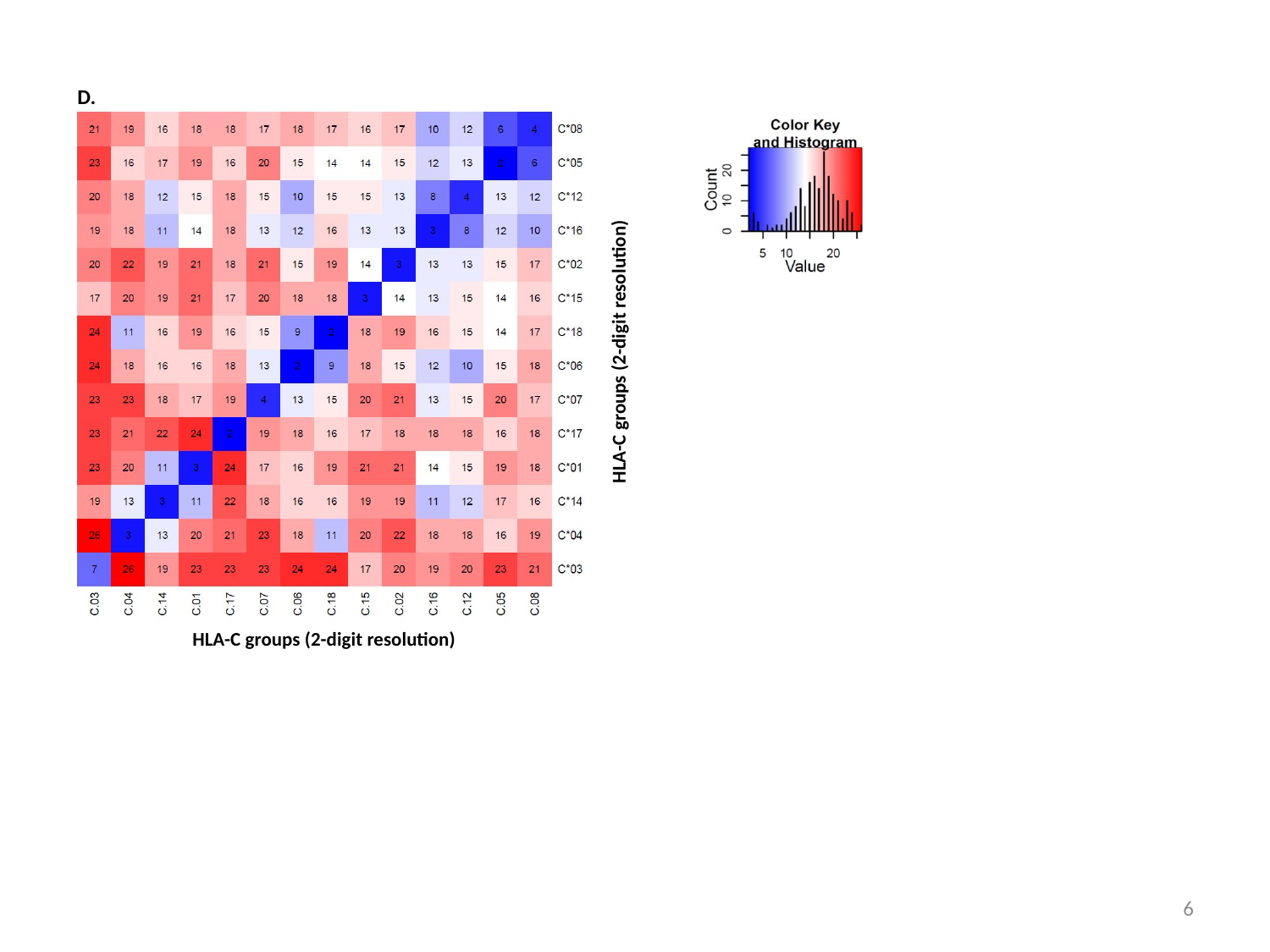

D.
HLA-C groups (2-digit resolution)
HLA-C groups (2-digit resolution)
6

## Slide 7
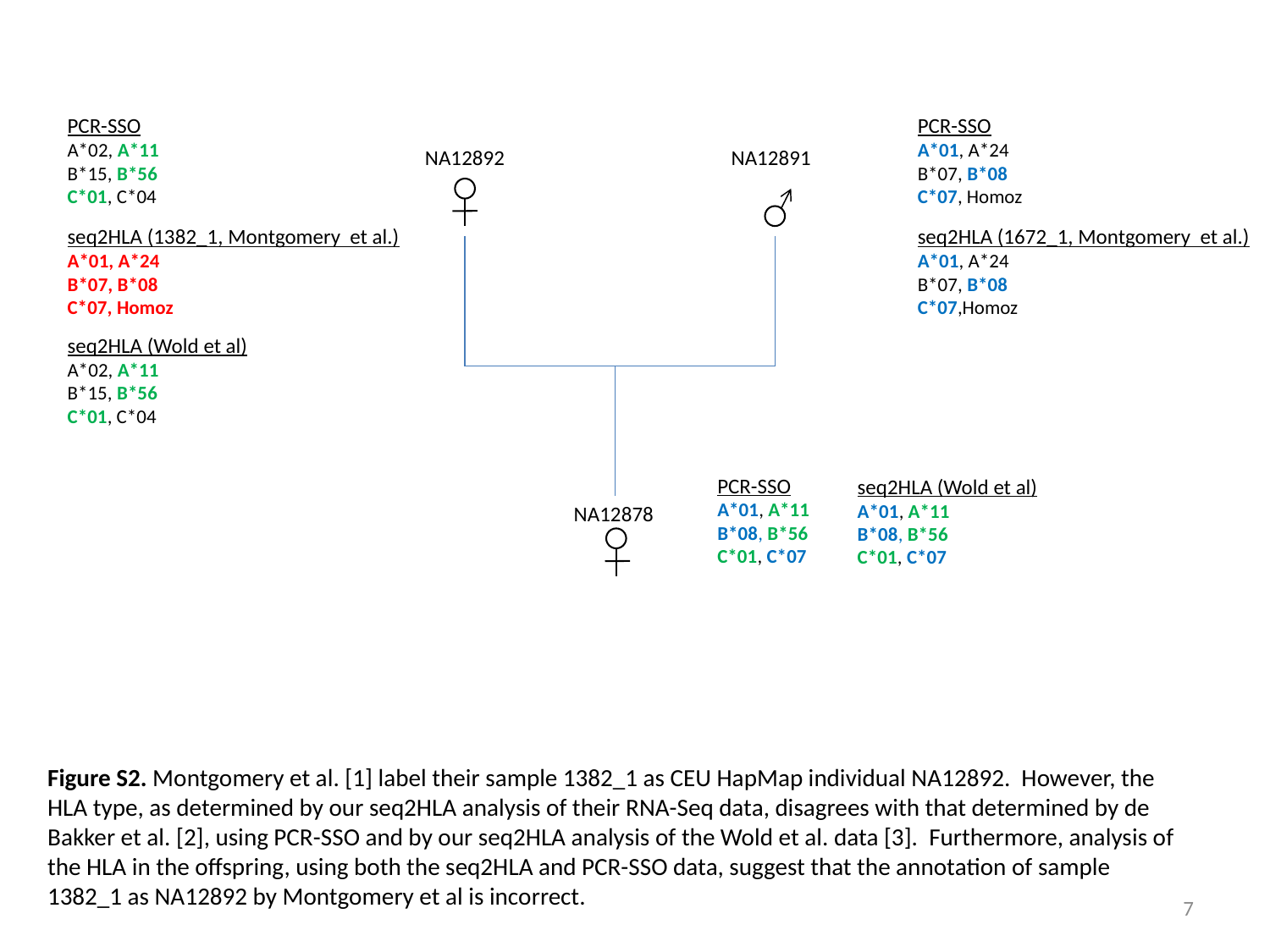

PCR-SSO
A*02, A*11
B*15, B*56
C*01, C*04
PCR-SSO
A*01, A*24
B*07, B*08
C*07, Homoz
NA12892
NA12891
seq2HLA (1382_1, Montgomery et al.)
A*01, A*24
B*07, B*08
C*07, Homoz
seq2HLA (1672_1, Montgomery et al.)
A*01, A*24
B*07, B*08
C*07,Homoz
seq2HLA (Wold et al)
A*02, A*11
B*15, B*56
C*01, C*04
PCR-SSO
A*01, A*11
B*08, B*56
C*01, C*07
seq2HLA (Wold et al)
A*01, A*11
B*08, B*56
C*01, C*07
NA12878
Figure S2. Montgomery et al. [1] label their sample 1382_1 as CEU HapMap individual NA12892. However, the HLA type, as determined by our seq2HLA analysis of their RNA-Seq data, disagrees with that determined by de Bakker et al. [2], using PCR-SSO and by our seq2HLA analysis of the Wold et al. data [3]. Furthermore, analysis of the HLA in the offspring, using both the seq2HLA and PCR-SSO data, suggest that the annotation of sample 1382_1 as NA12892 by Montgomery et al is incorrect.
7

## Slide 8
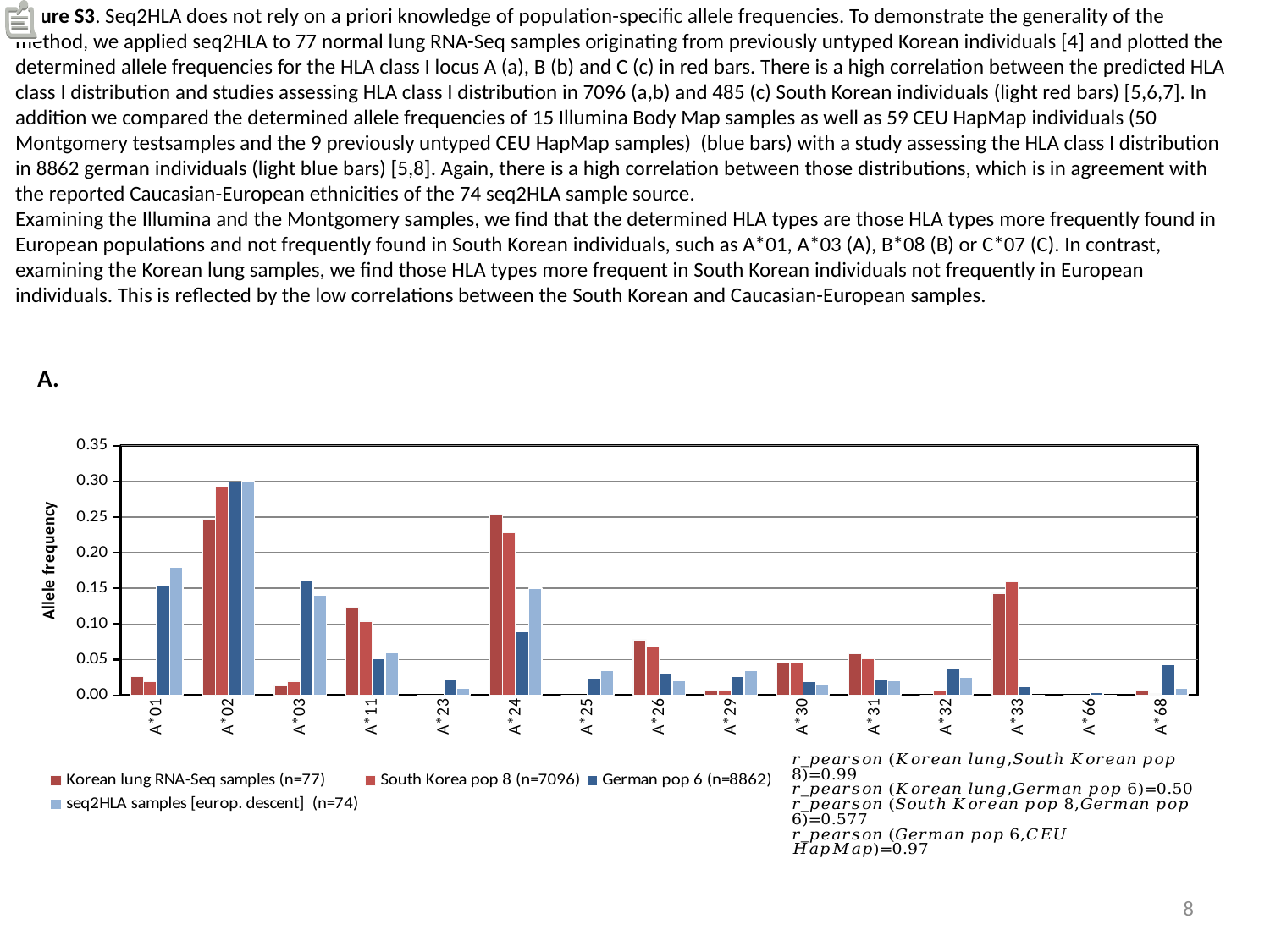

Figure S3. Seq2HLA does not rely on a priori knowledge of population-specific allele frequencies. To demonstrate the generality of the method, we applied seq2HLA to 77 normal lung RNA-Seq samples originating from previously untyped Korean individuals [4] and plotted the determined allele frequencies for the HLA class I locus A (a), B (b) and C (c) in red bars. There is a high correlation between the predicted HLA class I distribution and studies assessing HLA class I distribution in 7096 (a,b) and 485 (c) South Korean individuals (light red bars) [5,6,7]. In addition we compared the determined allele frequencies of 15 Illumina Body Map samples as well as 59 CEU HapMap individuals (50 Montgomery testsamples and the 9 previously untyped CEU HapMap samples) (blue bars) with a study assessing the HLA class I distribution in 8862 german individuals (light blue bars) [5,8]. Again, there is a high correlation between those distributions, which is in agreement with the reported Caucasian-European ethnicities of the 74 seq2HLA sample source.
Examining the Illumina and the Montgomery samples, we find that the determined HLA types are those HLA types more frequently found in European populations and not frequently found in South Korean individuals, such as A*01, A*03 (A), B*08 (B) or C*07 (C). In contrast, examining the Korean lung samples, we find those HLA types more frequent in South Korean individuals not frequently in European individuals. This is reflected by the low correlations between the South Korean and Caucasian-European samples.
A.
[unsupported chart]

## Slide 9
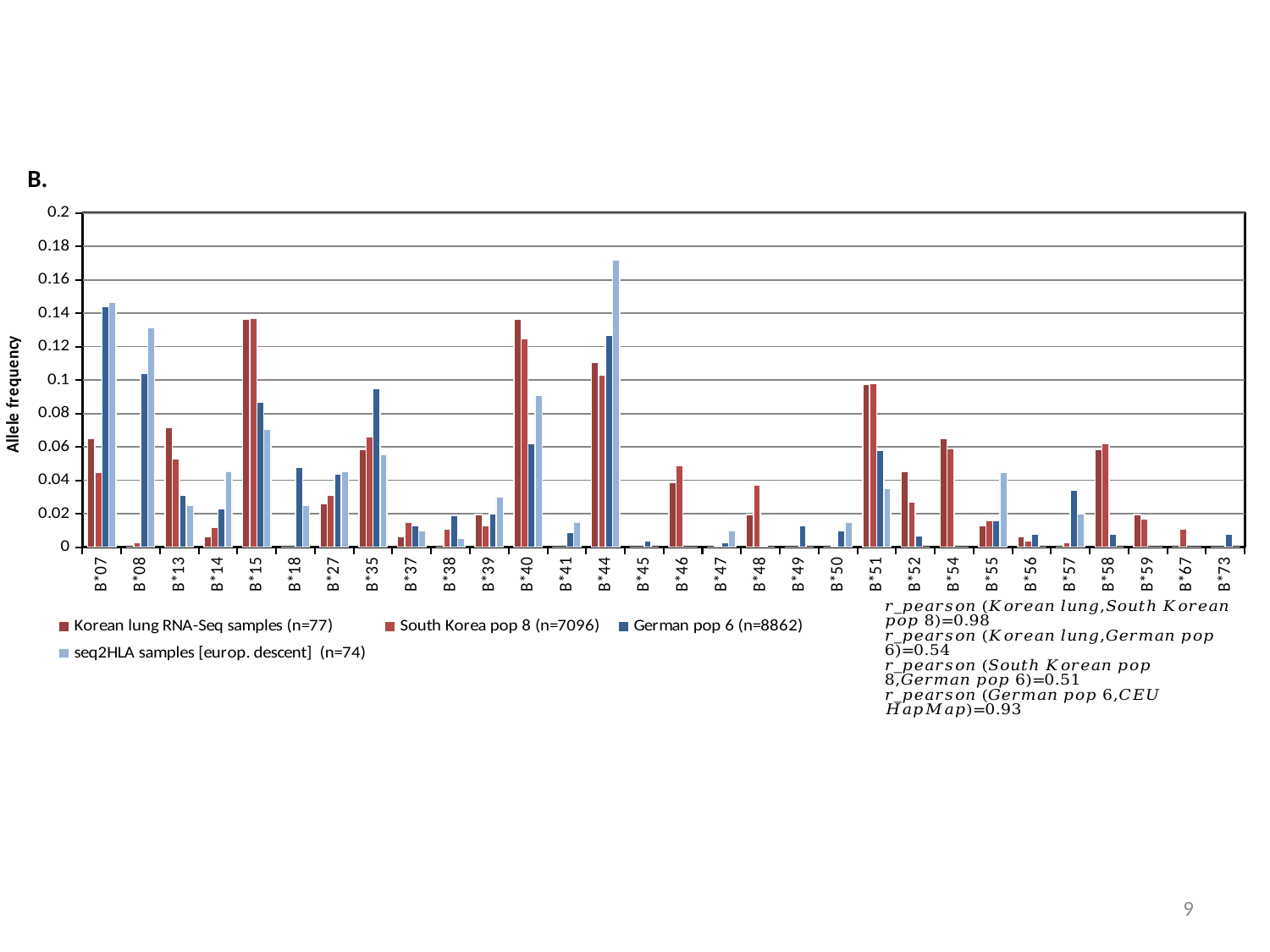

B.
### Chart
| Category | Korean lung RNA-Seq samples (n=77) | South Korea pop 8 (n=7096) | German pop 6 (n=8862) | seq2HLA samples [europ. descent] (n=74) |
|---|---|---|---|---|
| B*07 | 0.06493506493506493 | 0.045 | 0.144 | 0.14646464646464646 |
| B*08 | 0.0 | 0.003 | 0.104 | 0.13131313131313133 |
| B*13 | 0.07142857142857142 | 0.053 | 0.031 | 0.025252525252525252 |
| B*14 | 0.006493506493506494 | 0.012 | 0.023 | 0.045454545454545456 |
| B*15 | 0.13636363636363635 | 0.137 | 0.087 | 0.0707070707070707 |
| B*18 | 0.0 | 0.0 | 0.048 | 0.025252525252525252 |
| B*27 | 0.025974025974025976 | 0.031 | 0.044 | 0.045454545454545456 |
| B*35 | 0.05844155844155844 | 0.066 | 0.095 | 0.05555555555555555 |
| B*37 | 0.006493506493506494 | 0.015 | 0.013 | 0.010101010101010102 |
| B*38 | 0.0 | 0.011 | 0.019 | 0.005050505050505051 |
| B*39 | 0.01948051948051948 | 0.013 | 0.02 | 0.030303030303030304 |
| B*40 | 0.13636363636363635 | 0.125 | 0.062 | 0.09090909090909091 |
| B*41 | 0.0 | 0.0 | 0.009 | 0.015151515151515152 |
| B*44 | 0.11038961038961038 | 0.103 | 0.127 | 0.1717171717171717 |
| B*45 | 0.0 | 0.0 | 0.004 | 0.0 |
| B*46 | 0.03896103896103896 | 0.049 | 0.0 | 0.0 |
| B*47 | 0.0 | 0.001 | 0.003 | 0.01 |
| B*48 | 0.01948051948051948 | 0.037 | 0.001 | 0.0 |
| B*49 | 0.0 | 0.0 | 0.013 | 0.0 |
| B*50 | 0.0 | 0.001 | 0.01 | 0.015 |
| B*51 | 0.09740259740259741 | 0.098 | 0.058 | 0.035 |
| B*52 | 0.045454545454545456 | 0.027 | 0.007 | 0.0 |
| B*54 | 0.06493506493506493 | 0.059 | 0.0 | 0.0 |
| B*55 | 0.012987012987012988 | 0.016 | 0.016 | 0.045 |
| B*56 | 0.006493506493506494 | 0.004 | 0.008 | 0.0 |
| B*57 | 0.0 | 0.003 | 0.034 | 0.02 |
| B*58 | 0.05844155844155844 | 0.062 | 0.008 | 0.0 |
| B*59 | 0.01948051948051948 | 0.017 | 0.0 | 0.0 |
| B*67 | 0.0 | 0.011 | 0.0 | 0.0 |
| B*73 | 0.0 | 0.0 | 0.008 | 0.0 |

## Slide 10
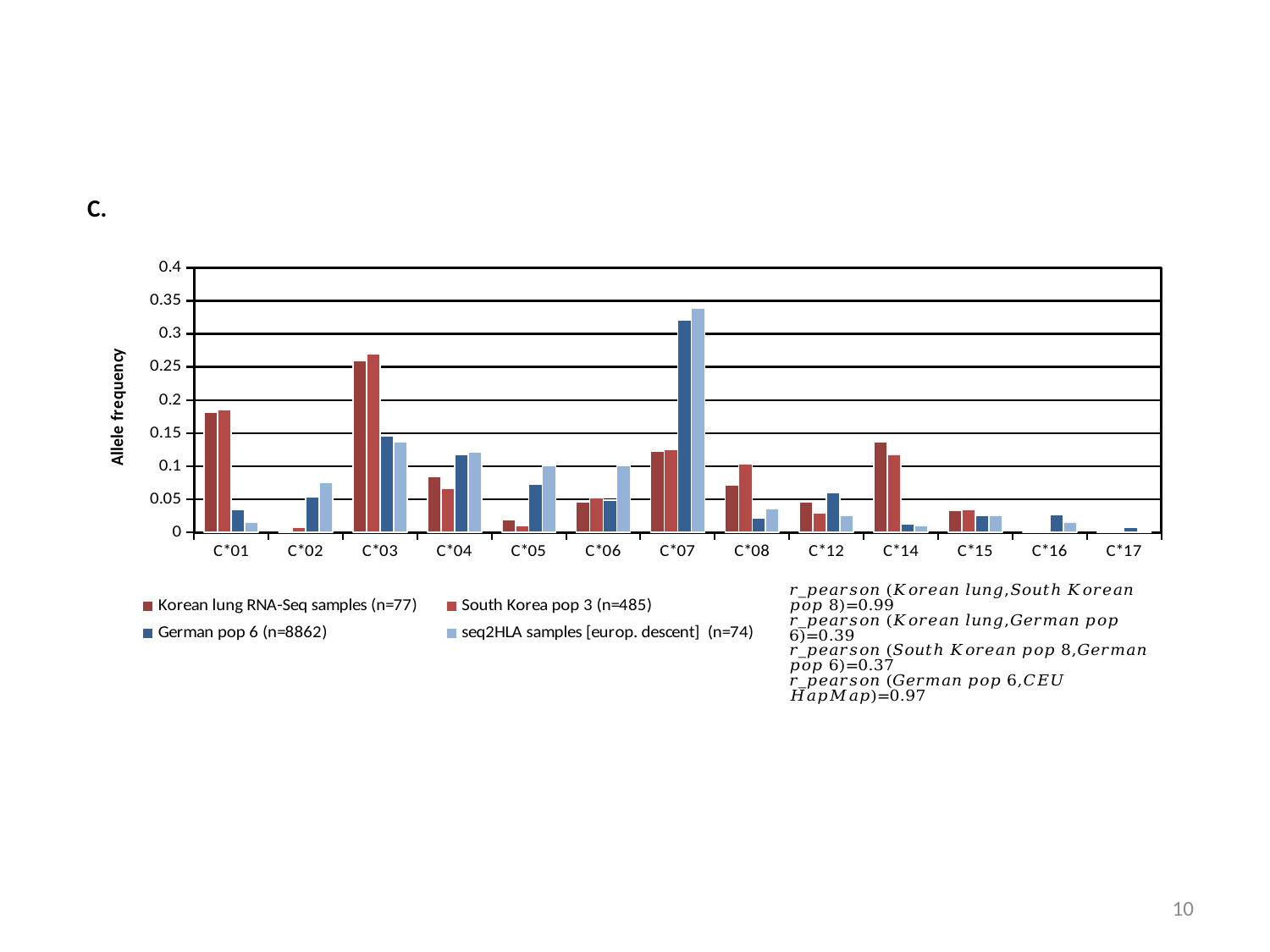

C.
### Chart
| Category | Korean lung RNA-Seq samples (n=77) | South Korea pop 3 (n=485) | German pop 6 (n=8862) | seq2HLA samples [europ. descent] (n=74) |
|---|---|---|---|---|
| C*01 | 0.18181818181818182 | 0.185 | 0.035 | 0.015151515151515152 |
| C*02 | 0.0 | 0.008 | 0.054 | 0.07575757575757576 |
| C*03 | 0.2597402597402597 | 0.27 | 0.146 | 0.13636363636363635 |
| C*04 | 0.08441558441558442 | 0.066 | 0.117 | 0.12121212121212122 |
| C*05 | 0.01948051948051948 | 0.01 | 0.073 | 0.10101010101010101 |
| C*06 | 0.045454545454545456 | 0.052 | 0.049 | 0.10101010101010101 |
| C*07 | 0.12337662337662338 | 0.125 | 0.321 | 0.3383838383838384 |
| C*08 | 0.07142857142857142 | 0.104 | 0.022 | 0.03535353535353535 |
| C*12 | 0.045454545454545456 | 0.029 | 0.06 | 0.025252525252525252 |
| C*14 | 0.13636363636363635 | 0.117 | 0.012 | 0.010101010101010102 |
| C*15 | 0.032467532467532464 | 0.034 | 0.026 | 0.025252525252525252 |
| C*16 | 0.0 | 0.0 | 0.027 | 0.015151515151515152 |
| C*17 | 0.0 | 0.0 | 0.008 | 0.0 |

## Slide 11
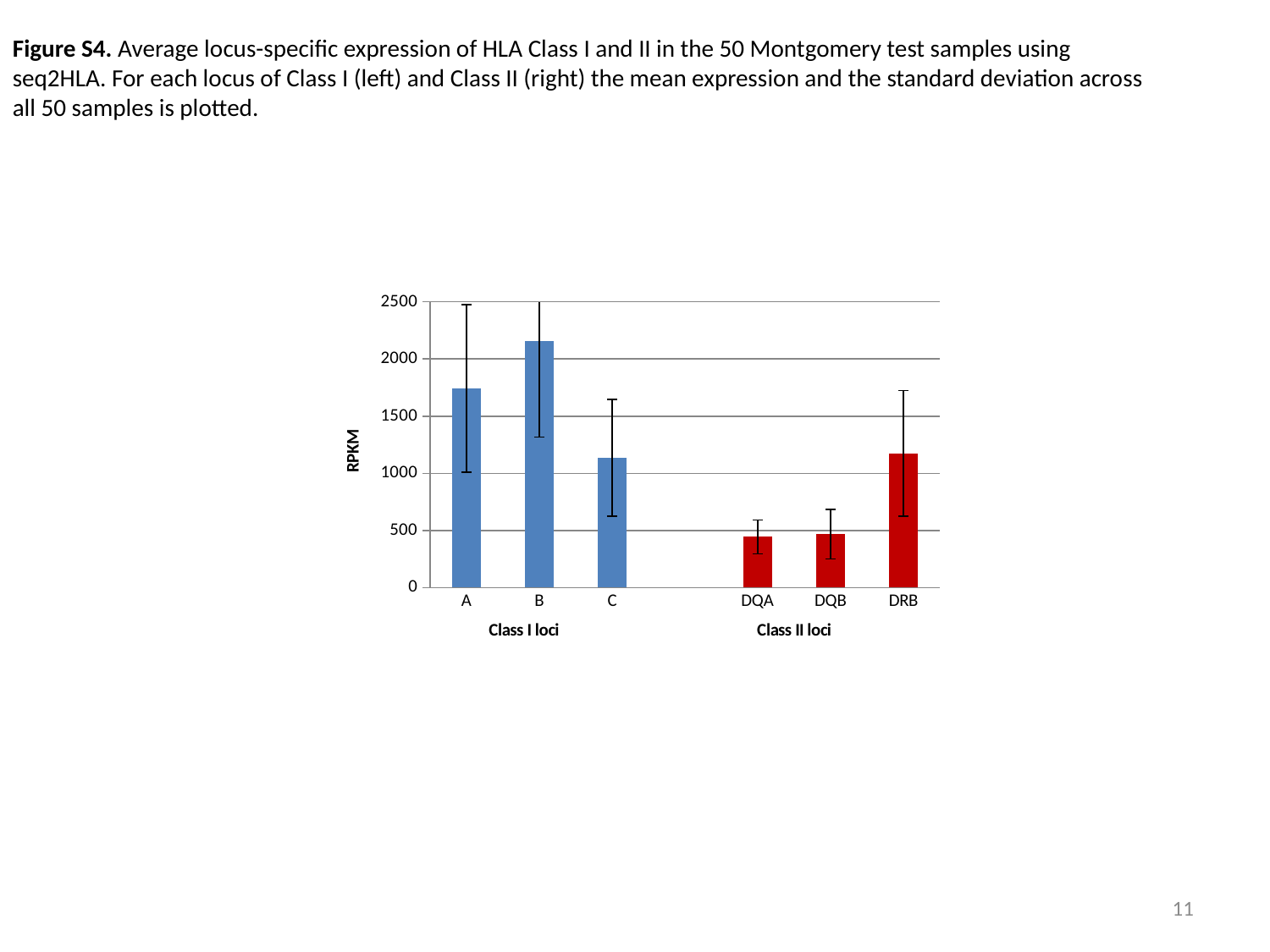

Figure S4. Average locus-specific expression of HLA Class I and II in the 50 Montgomery test samples using seq2HLA. For each locus of Class I (left) and Class II (right) the mean expression and the standard deviation across all 50 samples is plotted.
### Chart
| Category | |
|---|---|
| A | 1740.9554 |
| B | 2160.1077999999998 |
| C | 1135.9346 |
| | None |
| DQA | 444.319 |
| DQB | 469.1716 |
| DRB | 1174.9352000000001 |11

## Slide 12
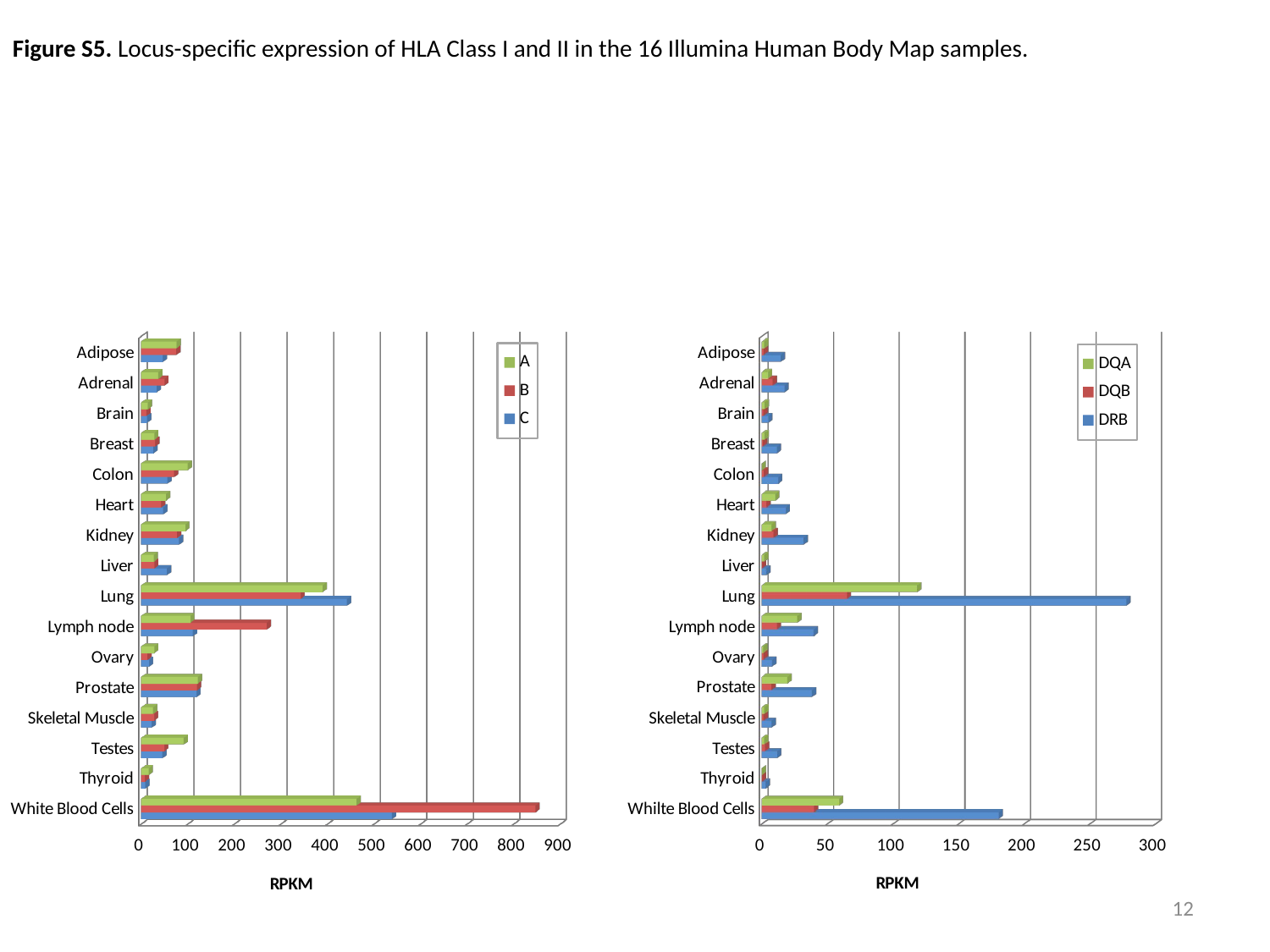

Figure S5. Locus-specific expression of HLA Class I and II in the 16 Illumina Human Body Map samples.
[unsupported chart]
[unsupported chart]
12

## Slide 13
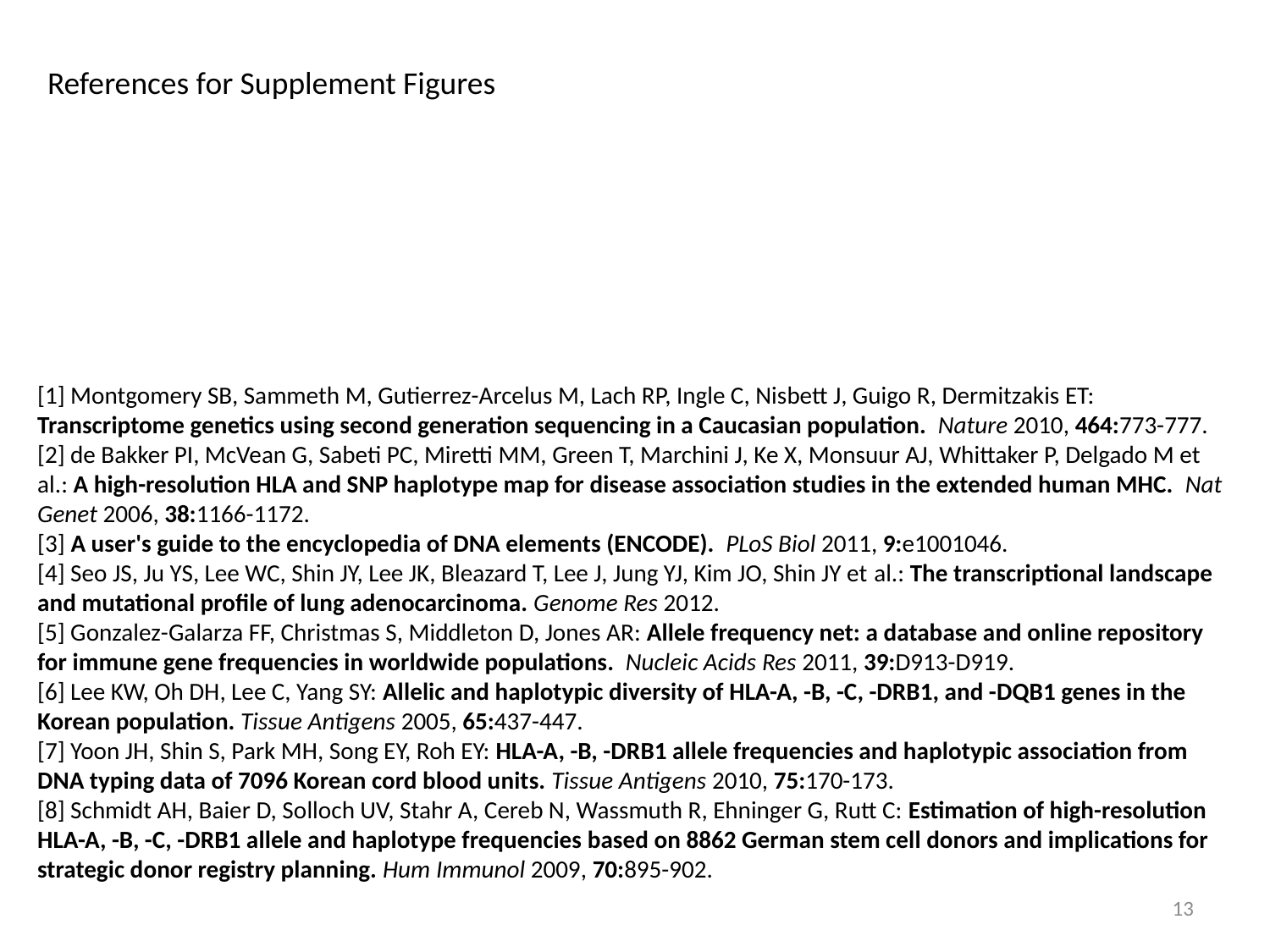

References for Supplement Figures
[1] Montgomery SB, Sammeth M, Gutierrez-Arcelus M, Lach RP, Ingle C, Nisbett J, Guigo R, Dermitzakis ET: Transcriptome genetics using second generation sequencing in a Caucasian population. Nature 2010, 464:773-777.
[2] de Bakker PI, McVean G, Sabeti PC, Miretti MM, Green T, Marchini J, Ke X, Monsuur AJ, Whittaker P, Delgado M et al.: A high-resolution HLA and SNP haplotype map for disease association studies in the extended human MHC. Nat Genet 2006, 38:1166-1172.
[3] A user's guide to the encyclopedia of DNA elements (ENCODE). PLoS Biol 2011, 9:e1001046.
[4] Seo JS, Ju YS, Lee WC, Shin JY, Lee JK, Bleazard T, Lee J, Jung YJ, Kim JO, Shin JY et al.: The transcriptional landscape and mutational profile of lung adenocarcinoma. Genome Res 2012.
[5] Gonzalez-Galarza FF, Christmas S, Middleton D, Jones AR: Allele frequency net: a database and online repository for immune gene frequencies in worldwide populations. Nucleic Acids Res 2011, 39:D913-D919.
[6] Lee KW, Oh DH, Lee C, Yang SY: Allelic and haplotypic diversity of HLA-A, -B, -C, -DRB1, and -DQB1 genes in the Korean population. Tissue Antigens 2005, 65:437-447.
[7] Yoon JH, Shin S, Park MH, Song EY, Roh EY: HLA-A, -B, -DRB1 allele frequencies and haplotypic association from DNA typing data of 7096 Korean cord blood units. Tissue Antigens 2010, 75:170-173.
[8] Schmidt AH, Baier D, Solloch UV, Stahr A, Cereb N, Wassmuth R, Ehninger G, Rutt C: Estimation of high-resolution HLA-A, -B, -C, -DRB1 allele and haplotype frequencies based on 8862 German stem cell donors and implications for strategic donor registry planning. Hum Immunol 2009, 70:895-902.
13
